# *DRB2* Modulates Leaf Rolling by Regulating Accumulation of MicroRNAs Related to Leaf Development in Rice

**DOI:** 10.3390/ijms231911147

**Published:** 2022-09-22

**Authors:** Zhaodi Yuan, Jihong Pan, Congping Chen, Yulin Tang, Hongshan Zhang, Jia Guo, Xiaorong Yang, Longfei Chen, Chunyan Li, Ke Zhao, Qian Wang, Bin Yang, Changhui Sun, Xiaojian Deng, Pingrong Wang

**Affiliations:** 1 State Key Laboratory of Crop Gene Exploration and Utilization in Southwest China, Sichuan Agricultural University, Chengdu 611130, China; 2 Rice Research Institute, Sichuan Agricultural University, Chengdu 611130, China

**Keywords:** rice (*Oryza sativa*), leaf rolling, bulliform cell, microRNA, *DRB2*, leaf development

## Abstract

As an important agronomic trait in rice (*Oryza sativa*), moderate leaf rolling helps to maintain the erectness of leaves and minimize shadowing between leaves, leading to improved photosynthetic efficiency and grain yield. However, the molecular mechanisms underlying rice leaf rolling still need to be elucidated. Here, we isolated a rice mutant, *rl89*, showing adaxially rolled leaf phenotype due to decreased number and size of bulliform cells. We confirmed that the *rl89* phenotypes were caused by a single nucleotide substitution in *OsDRB2* (*LOC_Os10g33970*) gene encoding DOUBLE-STRANDED RNA-BINDING2. This gene was constitutively expressed, and its encoded protein was localized to both nucleus and cytoplasm. Yeast two-hybrid assay showed that OsDRB2 could interact with DICER-LIKE1 (DCL1) and OsDRB1-2 respectively. qRT-PCR analysis of 29 related genes suggested that defects of the *OsDRB2*-miR166-*OsHBs* pathway could play an important role in formation of the rolled leaf phenotype of *rl89*, in which *OsDRB2* mutation reduced miR166 accumulation, resulting in elevated expressions of the class III homeodomain-leucine zipper genes (such as *OsHB1*, *3* and *5*) involved in leaf polarity and/or morphology development. Moreover, *OsDRB2* mutation also reduced accumulation of miR160, miR319, miR390, and miR396, which could cause the abnormal leaf development in *rl89* by regulating expressions of their target genes related to leaf development.

## 1. Introduction

Rice (*Oryza sativa*) is one of the most important food crops in the world, and how to increase grain yield has been the focus of rice research for a long time. The leaf is the major organ of plant photosynthesis, and moderate rolling of rice leaves helps to maintain the erectness of leaves and minimize shadowing between leaves, which can improve light acceptance and gas exchange and then increase the yield [1,2,3]. However, severe leaf rolling can lead to growth retardation, developmental defects, and yield reduction [4]. Therefore, isolating mutants with moderately rolled leaves and identifying the leaf rolling-related genes will be beneficial for breeding the super-high-yield rice varieties with the desired architecture [5].

Most leaf rolling phenotypes are resulted from abnormal morphology, number, size, or distribution of the bulliform cells (BCs) on the adaxial surface of leaf blades in rice [6]. To date, some rice genes have been identified that regulate leaf rolling by altering the number and/or size of bulliform cells [7]. For instance, knock-out of *Rice outermost cell-specific gene5* (*Roc5*), encoding a class-IV homeodomain-leucine zipper (HD-Zip IV) transcription factor, led to abaxial leaf rolling by increasing number and size of BCs on the adaxial surface, whereas overexpression of *Roc5* caused adaxial rolling by reducing the BC number and size [8]. Semi-dominant mutant *upward rolled leaf 1* (*url1*) showed adaxially rolled leaves due to a decrease in number and size of BCs, which was resulted from a single-base substitution in *URL1* gene encoding the HD-Zip IV family member Roc8 [7]. *SEMIROLLED LEAF1* (*SRL1*) encodes a putative glycosylphosphatidylinositol-anchored protein, and its mutant *srl1* exhibited adaxially rolled leaves due to increased number of bulliform cells at the adaxial cell layers [2]. Meanwhile, many other genes of rice modulate leaf rolling by regulating number, size, and arrangement of BCs, such as *ACL1* (*abaxially curled leaf 1*) and *ACL2*, *NAL2* (*narrow leaf 2*), and *NAL3*, *ZHD1* (*Zn-finger transcription factor*), *REL1* (*rolled and erect leaf 1*), and *REL2* [3,9,10,11,12]. In addition, several genes regulating secondary cell wall or cellulose formation also play important roles in determining leaf rolling by generally affecting the development of BCs, such as *RL14* (*Rolling-leaf14*), *SRL1/CLD1* (*curled leaf and dwarf 1*), *PSL1* (*PHOTO-SENSITIVE LEAF ROLLING 1*), *OsCSLD4* (*cellulose synthase-like D4*)/*NRL1* (*narrow and rolled leaf 1*), and *OsMYB103L* (*R2R3-MYB transcription factor*) [4,6,13,14,15,16]. Except BCs, some studies demonstrated that mesophyll cells, sclerenchyma cells, epidermal cells, and cuticular wax also play roles in the regulation of leaf rolling. For example, *SHALLOT-LIKE 1* (*SLL1*) encodes a SHAQKYF class MYB family transcription factor that belongs to the KANADI family, and its mutant (*sll1*) displayed extremely incurved leaves because of the defective development of sclerenchymatous cells on the abaxial side. Further study indicated that *SLL1* deficiency leads to abnormal programmed cell death of abaxial mesophyll cells and suppresses the development of abaxial features [17]. *CURLY FLAG LEAF* (*CFL1*) encoding a WW domain protein negatively regulated cuticle development by affecting the function of HDG1, an HD-Zip IV transcription factor, and loss-of-*CFL1*-function resulted in curly leaves whose epidermis were wrinkled and covered with less waxy papillae [18]. Nonetheless, the molecular mechanisms of leaf rolling still need to be elucidated.

MicroRNAs (miRNAs) are a class of 21–22 nucleotide small noncoding RNAs that negatively regulate target gene expression through complementary base pairing at the post-transcriptional level [19]. In plants, hairpin-containing primary miRNAs (Pri-miRNAs) are processed into mature strand miRNAs by DICER-LIKE1 (DCL1) bound to SERRATE (SE) and either DOUBLE-STRANDED RNA-BINDING1 (DRB1) or DOUBLE-STRANDED RNA-BINDING2 (DRB2). Subsequently, the strand miRNA is protected from uridylation through HUA ENHANCER1 (HEN1), and then loaded by ARGONAUTE1 (AGO1) to form miRNA-induced silencing complex (miRISC), which repress their target transcripts mainly in two ways: cleavage (DRB1) and translational repression (DRB1 and DRB2) [20,21,22]. On the other hand, it was showed that mutations of Arabidopsis *DCL1*, *SE*, *DRB1,* and *HEN1* genes reduced miRNA accumulation and increased target transcription [23], and their mutants *dcl1*, *drb1*, *se*, *se-2*, *se-3,* and *hen1* displayed many similar developmental defects [24,25,26,27]. Meanwhile, it was indicated that Arabidopsis *DRB2* gene could repress *DRB1* expression at the transcriptional level, and elevated expression levels of *DRB1* and *DRB2* could partially compensate for the loss of DRB2 and DRB1 activities, respectively, in which the inter-regulation between *DRB1* and *DRB2* showed the key role played by *DRB2* in plant growth and development [21,28]. So far, *DRB2* genes have been characterized in dicotyledonous plants, such as Arabidopsis [28] and soybean (*Glycine max*) [29]. The T-DNA insertion mutant of Arabidopsis *DRB2* showed rosette leaves with ovoid and that were flatter and darker in color [28]. However, no *DRB2* gene has been identified from monocotyledonous plants, and the function of *DRB2* gene remains elusive in rice.

In this study, we isolated a leaf-rolling mutant, *rl89*, in rice. The mutant showed adaxially rolled leaf phenotype due to decreased number and size of bulliform cells. MutMap cloning, genetic complementation and CRISPR/Cas9 knockout assays demonstrated that a single nucleotide mutation in the *OsDRB2* gene accounted for the mutant phenotype of *rl89*. This gene is constitutively expressed, and its encoded protein was localized to both nucleus and cytoplasm. In addition, we investigated transcriptional changes of 29 related genes in *rl89* by qRT-PCR, revealing that *OsDRB2* mutation causes leaf rolling by reducing accumulation of MicroRNAs related to leaf polarity and/or leaf morphology development.

## 2. Results

### 2.1. Phenotypes of rl89 Mutant

**Figure 1:**
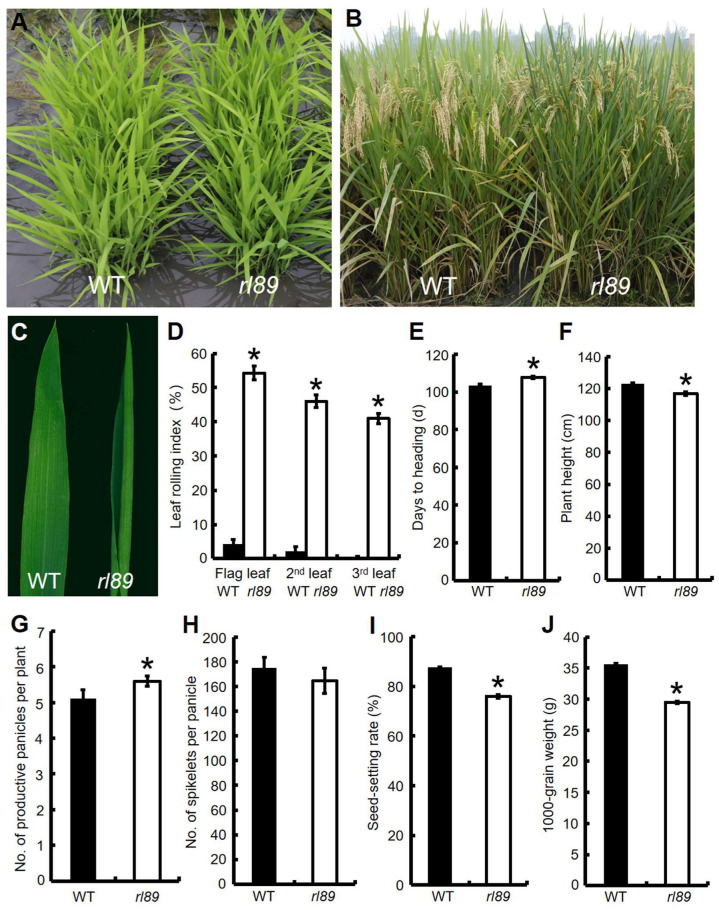
Comparison of plant phenotypes and major agronomical traits between *rl89* mutant and its wild type (WT). (**A**) Seedlings at the four-leaf stage. (**B**) Plants in the field at the grain-filling stage. (**C**) Flag leaf at the heading stage. (**D**) Leaf rolling indexes (LRIs) of the top three leaves (the flag, 2nd, and 3rd leaves from the top of main stem of a single plant) at the heading stage. (**E**) Days to heading. (**F**) Plant height. (**G**) No. of productive panicles per plant. (**H**) No. of spikelets per panicle. (**I**) Seed setting rate. (**J**) 1000-grain weight. Bars represent standard deviations (SDs) of three independent experiments in (**D**–**J**), respectively. * indicates statistically significant difference compared to the wild type at *p* < 0.05.

The *rl89* mutant was isolated from *indica* rice restorer line Lehui188 (188R) by ethyl methanesulfonate (EMS) mutagenesis. The mutant was normal at seedling stage and started to show adaxially rolled leaves when the twelfth leaves emerged (Figure 1A–C). At the heading stage, leaf-rolling index values (LRIs) of the last emerged three leaves (the top three leaves) were 41.0% to 54.3%, whereas the corresponding wild-type leaves were almost flat (Figure 1D). On the other hand, the mutant grew slowly from tillering stage to booting stage, and its heading stage was delayed by about 5 days (Figure 1E). At maturity, except number of spikelets per panicle (Figure 1H), major agronomic traits of *rl89* were significantly affected. For instance, its plant height, seed setting rate, and 1000-grain weight were reduced by 5.1%, 11.6%, and 17.3%, respectively (Figure 1F,I,J), but the number of productive panicles per plant was increased by 9.8% (Figure 1G), compared with those of the wild type. In addition, the *rl89* mutant also exhibits other abnormal phenotypes, such as significant reduction in length of panicle, length, and width of grain (Figure S1).

To identify physiological characteristics of the mutant, photosynthetic pigment contents were measured, revealing that contents of total chlorophyll (Chl), Chl *a* and Chl *b* in the leaves of *rl89* were increased to some extent, compared with those in the wild type (Figure S2A). Nonetheless, its net photosynthetic rate, stomatal conductance, and transpiration rate were significantly decreased, compared to the wild type (Figure S2B–D).

**Figure 2:**
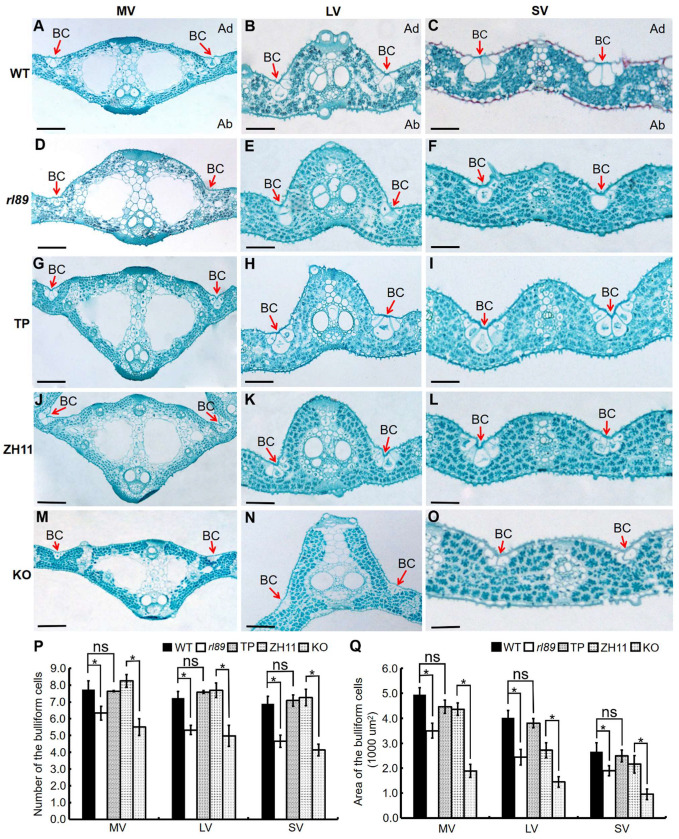
Number, size, and area of bulliform cells in midrib, large veins, and small veins of flag leaves at the booting stage. Cross sections of the middle sections of flag leaves in the wild type (WT, (**A**–**C**)), the *rl89* mutant (**D**–**F**), the *OsDRB2* transgenic plants (TP, (**G**–**I**)), the control variety Zhonghua 11 (ZH11, (**J**–**L**)), and the *OsDRB2* knockout mutant (KO, (**M**–**O**)). MV, midrib vein; LV, large vein; SV, small vein; Ad, adaxial; Ab, abaxial. Red arrows indicate bulliform cell (BC). Black bars indicate 100 μm. (**P**,**Q**) Statistical analysis of bulliform cells. Error bar represents the SD (*n* > 6). * and ns indicate statistically significant and non-significant differences, respectively, at *p* < 0.05.

To explore the formation of leaf rolling, paraffin cross-sectioning of flag leaves was performed at the booting stage. Typically, the number and size of bulliform cells in the midrib, large and small veins of *rl89* mutant were obviously decreased (Figure 2A–F,P). Consequently, total area of bulliform cells adjoining the midrib, large veins, and small veins were significantly reduced by 20.0%, 39.4%, and 28.7%, respectively in *rl89*, compared with those in the wild type (Figure 2Q). This result implied that the adaxial leaf rolling could be resulted from the reduced number and size of bulliform cells in the mutant.

### 2.2. The rl89 Locus Was Mapped to a Putative Gene Encoding DRB2

For genetic analysis of the mutant phenotype, *rl89* was crossed with the wild-type parent 188R. The F_1_ plants from the cross all displayed a normal flat-leaf phenotype. The resulting F_2_ population showed segregation of the normal flat-leaf phenotype and the leaf rolling phenotype after the twelfth leaf stage, with a ratio of 3:1 (χ^2^ < χ^2^_0.05_ = 3.84, *p* > 0.05, Table S1). The result suggested that the leaf rolling phenotype of *rl89* is controlled by a single recessive nuclear gene.

**Figure 3:**
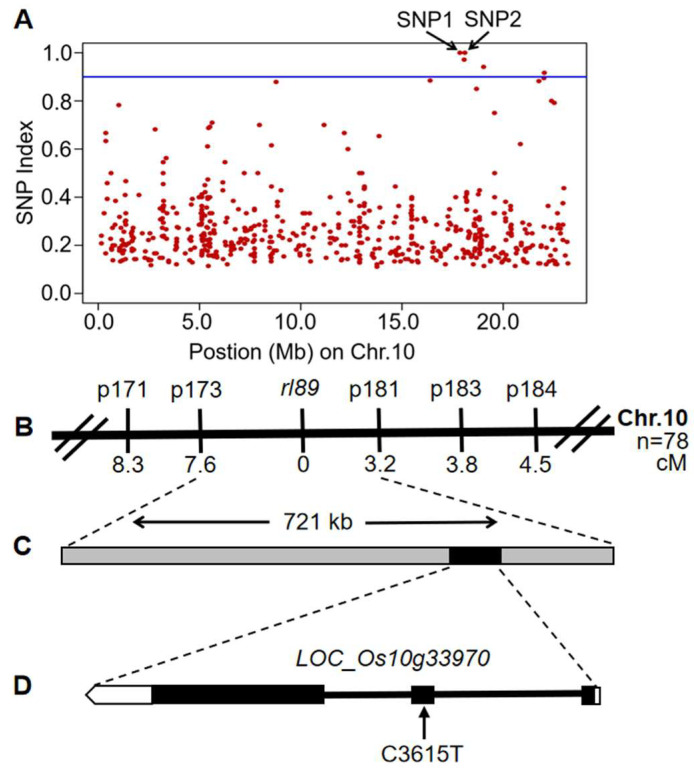
Identification of the causal SNP of the *rl89* mutant using MutMap approach and molecular markers. (**A**) SNP index plots for homozygous mutant plants from (*rl89*/188R) F_2_ population. (**B**) The *rl89* locus was mapped to a region between the InDel markers p173 and p181 on the long arm of rice chromosome 10. (**C**) The physical distance between InDel marker p173 and p181 is 721 kb, among which the black box represents the candidate gene *LOC_Os10g33970*. (**D**) *LOC_Os10g33970* is composed of 3 exons and 2 introns, and a single nucleotide C-to-T substitution occurred at position 3615 of its coding region in the *rl89* mutant. The black squares represent exons, the black lines represent introns, and the black arrow indicates the mutated nucleotide.

Next, the high-throughput sequencing was used to identify the candidate gene with 30 mutant plants showing typical leaf rolling phenotype from the above (*rl89* × 188R) F_2_ population. The MutMap analysis revealed two single-nucleotide polymorphisms (SNP1 and SNP2) with SNP index (a ratio between the number of reads of a mutant SNP and the total number of reads corresponding to the SNP) of 1.0, which all are located on the long arm of chromosome 10 (Figure 3A and Figure S3). SNP1 is located in the intron region of *LOC_Os10g33770* encoding a putative protein containing importin-beta N-terminal domain, whereas SNP2 is located in the exon region of *LOC_Os10g33970* encoding a putative protein containing double-stranded RNA binding motif and could lead to an amino acid change from Ala-146 to Val at the encoded protein. Furthermore, blastP in National Center for Biotechnology Information database (http://www.ncbi.nlm.nih.gov/BLAST/, accessed on 30 September 2017) suggested that the protein encoded by *LOC_Os10g33970* is homologous to DRB2 of Arabidopsis, and the Ala-146 in this protein is highly conserved in all species. Therefore, *LOC_Os10g33970* was firstly considered as the candidate gene of *rl89,* and tentatively designated as *OsDRB2* gene.

After that, the genetic linkage map of *rl89* locus was constructed by using insertion/deletion (InDel) markers (Table S2) and 78 mutant plants showing typical leaf-rolling phenotype from the (*rl89* × *japonica* cv. Nipponbare) F_2_ population. As a result, the *rl89* locus was mapped to a 721-kb interval between InDel markers p173 and p181, at genetic distances of 7.6 cM and 3.2 cM, respectively, and *OsDRB2* (*LOC_Os10g33970*) gene was just located in this region (Figure 3B, C). Then, we amplified and sequenced the *OsDRB2* gene in *rl89* mutant and its wild-type parent 188R (Table S3), and the result showed that a single nucleotide cytosine (C) at position 3615 in the genomic sequence was substituted by thymine (T) in *rl89* mutant, leading to an amino acid change from Ala-146 to Val at the encoded protein (Figure 3D). Meanwhile, we sequenced cDNA of this gene from *rl89* mutant and its wild type using reverse transcription (RT)-PCR and confirmed the point mutation mentioned above. Therefore, we took a step forward to verify *OsDRB2* as the candidate gene of *rl89*.

Sequencing revealed that the *OsDRB2* gene comprises three exons and two introns with 6191-bp DNA and 1545-bp cDNA, respectively. It encodes a 514-amino acid protein with a molecular weight of approximately 56 kDa. According to multiple amino acid sequence alignment, OsDRB2 has a similarity to its homologues in monocotyledonous plants sorghum (*Sorghum bicolor*), maize (*Zea mays*), and barley (*Hordeum vulgare*), and dicotyledonous plants tobacco (*Nicotiana attenuate*), soybean (*Glycine max*), and *Arabidopsis thaliana* (DRB2), with identity of 73.0, 72.0, 69.1, 66.0, 65.7%, and 63.9%, respectively (Figure S4). Phylogenetic analysis suggested that OsDRB2 has closer relationship with the homologues from sorghum and maize than those from other species (Figure S5).

### 2.3. Functional Confirmation of OsDRB2 through Complementation and Knockout Assays

**Figure 4:**
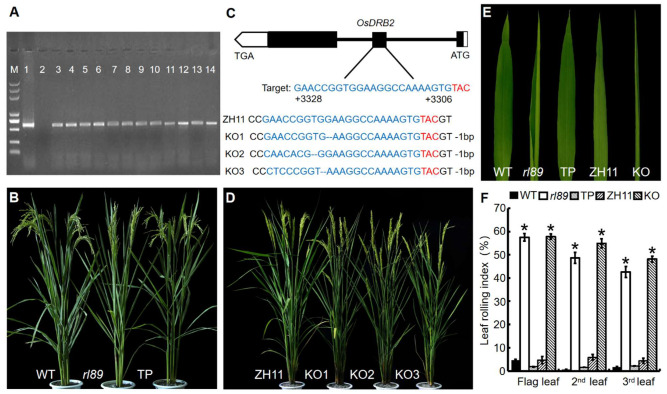
Complementation of the *rl89* mutant and knockout of the *OsDRB2* gene. (**A**) Identification of transgenic lines by PCR. M, DL-2000 plus Marker; 1, pCAMBIA1300-*OsDRB2* plasmid (PCR positive control); 2, *rl89* (PCR negative control); 3–14, positive transgenic plants (TP). (**B**) Phenotypes of the wild type (WT), *rl89,* and TP during the grain filling stage. (**C**) Schematic diagram of the target site in the *OsDRB2*. The 5’-UTR, 3’-UTR, exons, and introns are indicated by white rectangles, white pentagons, black rectangles, and black lines, respectively. The targeted site is labeled in blue uppercase letters, and the protospacer adjacent motif (PAM) sequences are highlighted in red. Sequence alignment of the *OsDRB2* target site was performed between the knockout mutants (KO, including KO1, KO2, and KO3) and the control variety Zhonghua 11 (ZH11) background. The deleted sequences are shown by blue hyphens, and the number of the deleted nucleotides is showed on the right. (**D**) Phenotypes of ZH11 and the knockout mutants during the grain filling stage. (**E**) Flag leaf phenotypes of WT, *rl89*, TP, ZH11, and KO at the heading stage. (**F**) LRIs of the top three leaves (the flag, 2nd and 3rd leaves from the top of main stem of a single plant) from WT, *rl89*, TP, ZH11, and KO at the heading stage. A error bar represents the SD of three independent experiments. Asterisks indicate statistically significant differences among WT, *rl89*, and TP, or between ZH11 and KO at *p* < 0.05.

In order to further confirm that the *rl89* leaf-rolling phenotype was caused by *OsDRB2* mutation, we carried out functional complementation experiment. First, the construct pCAMBIA1300-*OsDRB2* was generated, which carried a genomic fragment of 6191 bp comprising 2234 bp of the promoter region, 3362 bp of the entire *OsDRB2* sequence and 595 bp of the 3′-UTR region. Then, the resulting construct was introduced into the *rl89* mutant by *Agrobacterium*-mediated transformation. Finally, 12 positive transgenic lines were successfully obtained by identification with PCR test (Figure 4A). These positive transgenic lines all showed normal leaf shape as the wild type (Figure 4B,E,F), and their major agronomic traits are all similar to those of the wild type (Figure S6). Moreover, the transgenic lines expressing *OsDRB2* restored to normal morphology, number, and size of bulliform cell as the wild type (Figure 2G–I,P,Q). On the other hand, we generated *OsDRB2* knockout mutants (KO) through the CRISPR/Cas9 approach in the *japonica* cv. Zhonghua 11 (ZH11) background [30]. The result showed that seven independent positive transgenic mutants were obtained through sequencing analysis (Figure 4C). These KO mutants were normal at seedling stage, but their leaf-rolling and growth phenotypes at the late stage of growth look more severe than *rl89*, which could be because the *OsDRB2* gene completely lost function in these KO lines. Investigation of three KO lines showed that their LRIs of the top three leaves increased to 48.1% to 57.8% (Figure 4D–F). Meanwhile, the number and size of bulliform cells in their leaves were obviously reduced (Figure 2J–Q). In addition, most of their major agronomic traits were significantly affected (Figure S6). Therefore, both complementation and knockout assays confirmed that mutation in the *OsDRB2* gene can cause rolling leaves in rice and that a single nucleotide mutation in this gene is responsible for the mutant phenotype of *rl89*.

### 2.4. Subcellular Localization of OsDRB2 Protein

**Figure 5:**
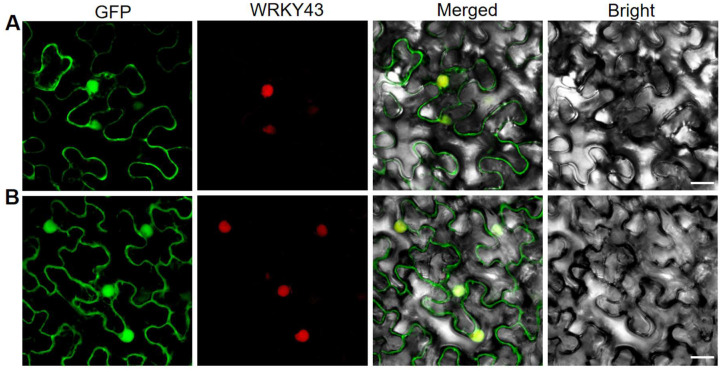
Subcellular localization of OsDRB2 protein. (**A**) GFP signals of the OsDRB2-eGFP fusion protein. (**B**) Empty vector eGFP without a specific targeting sequence. Green fluorescence shows GFP, red fluorescence indicates the fluorescence of nuclear marker gene *WRKY45*, yellow fluorescence indicates images with the two types of fluorescence merged, and bright field images show tobacco cells. Fluorescence signals were visualized using a laser-scanning confocal microscopy. Bars = 20 μm.

It was reported that Arabidopsis DRB2 was located in the cytoplasm and in the nucleus [31]. To confirm the localization of OsDRB2 in rice, we constructed the vector expressing the OsDRB2-GFP fusion protein. Then, the resulting pC2300-35S-*OsDRB2*-eGFP vector and the empty eGFP vector (a control) were co-transformed with the *WRKY45-*RFP vector (as a marker for nuclear) into tobacco cells, respectively. The result showed that green fluorescence from OsDRB2-GFP fusion protein was not only overlapped with red fluorescence from WRKY45, but also presented in cytoplasm (Figure 5A). As a control, the green fluorescence of eGFP was observed throughout the cell, including nucleus and cytoplasm (Figure 5B). This result suggested that OsDRB2 is indeed located in both nucleus and cytoplasm.

### 2.5. Expression Analyses of the OsDRB2 Gene

**Figure 6:**
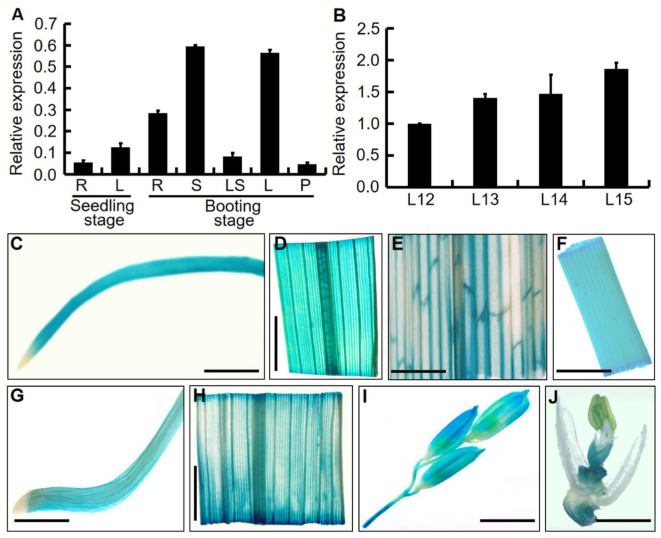
Expression pattern analysis of *OsDRB2*. (**A**) The spatiotemporal expression of *OsDRB2* in root (R), leaf blade (L), stem (S), leaf sheath (LS), and young panicle (P) of the wild type at seedling stage (three-leaf stage) and booting stage. (**B**) Expression levels of *OsDRB2* in leaf blades of the wild type at different developmental stages. L12, L13, L14, and L15 indicate the 12th, 13th, 14th, and 15th full-expanded leaves from the stem base, respectively (the 15th leaf was flag leaf). The expression level of *OsDRB2* in the 12th leaf was set to 1.0, and those at the other leaves were calculated accordingly. Error bars represent the standard deviations (SDs) of three independent experiments in (**A**,**B**). (**C**–**J**) Histochemical analysis of *OsDRB2* expression by GUS staining. *OsDRB2* was expressed in root and leaf blade at seedling stage (**C**,**D**), leaf sheath I, stem (**F**), root (**G**), leaf blade (**H**), young panicles and branches (**I**), and stamens and pistils (**J**) at booting stage. Black bar = 5 mm.

In order to explore the expression pattern of *OsDRB2*, its transcriptional levels in different tissues of the wild type were analyzed by qRT-PCR at the seedling stage and the booting stage, respectively. The results showed that *OsDRB2* was constitutively expressed in all tissues, including roots, stems, leaf blades, leaf sheaths, and young panicles. However, the expression levels of *OsDRB2* were variable in different tissues. More specifically, stems and leaf blades had the highest expression, followed by roots, leaf sheaths, and young panicles showed remarkably lower expression levels (Figure 6A). In addition, we tested the expression of *OsDRB2* in leaf blades at different stages (including the twelfth-, thirteen-, fourteen-, and fifteen-leaf stages). Expression levels in leaf blades remarkably increased with development, with the highest transcription level in the fifteen-leaf (flag leaf) (Figure 6B).

To further detect the expression pattern of *OsDRB2* gene, β-glucuronidase (GUS) staining was carried out with tissues and organs from transgenic plants transformed with pCAMBIA1391Z vector containing 2234-bp promoter sequence of *OsDRB2* gene and GUS reporter gene. As shown in Figure 6C–J, GUS staining was also visualized in all tissues, including roots and leaf blades at the seedling stage (Figure 6C,D), and leaf sheaths, stems, roots, leaf blades, and young panicles at the booting stage (Figure 6E–I), whereas not in anther, filament, and style (Figure 6J), which were basically consistent with the above results of qRT-PCR.

### 2.6. Protein Interaction of OsDRB2 with OsDCL1 and OsDRB1-2

**Figure 7:**
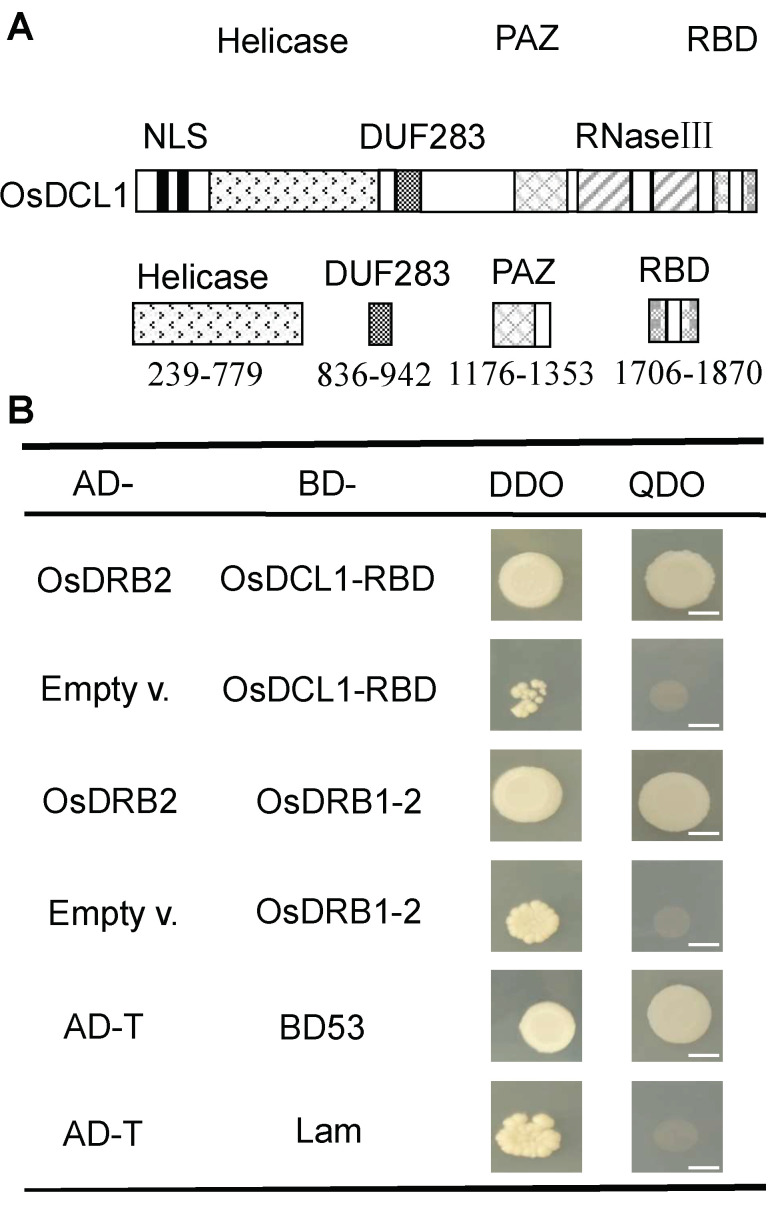
Interaction of OsDRB2 with OsDCL1-RBD and OsDRB1-2. (**A**) The structure diagram of DCL1 protein. Abbreviations of domains are as follows: NLS: nuclear localization signal; Helicase: DExD/H-box RNA helicase; DUF283: domain of unknown function 283; PAZ: Piwi/Argonaute/Zwille domain; RNase III: ribonuclease III; RBD: dsRNA-binding domains. (**B**) Yeast two-hybrid experiments showed the interactions of OsDRB2 with OsDCL1-RBD and OsDRB1-2 respectively. Interaction between pGADT7-T (AD-T) and pGBKT7-53 (BD53) was used as a positive control, and interaction between pGADT7-T (AD-T) and pGBKT7-Lam (Lam) was used as a negative control. Co-transformed yeast colonies were spotted on DDO (−Leu/−Trp) non-selective medium and QDO (−Leu/−Trp/−His/−Ade) selective medium, respectively. White bars indicate 5 mm.

In Arabidopsis, pull-down assay showed that miRNA processing protein DCL1, DRB1, and DRB2 can interact with each other [32]. In four rice DRB1 proteins homologous to Arabidopsis DRB1, OsDRB1-1, OsDRB1-2, and OsDRB1-4 were shown interaction with each other by yeast two-hybrid (Y2H) and BiFC assays [33]. However, the interactions of OsDRB2 with OsDCL1 and OsDRB1 have not been reported so far. So, we explored whether OsDRB2 interact with OsDCL1 and OsDRB1-2 by Y2H assay. First, the full-length cDNA sequence of *OsDRB2* was fused into GAL4 AD vector, and the *DCL1* cDNA fragment encoding its two C-terminal dsRBDs (DCL1-RBD, AA1706-1870) (Figure 7A) and the full-length *OsDRB1-2* cDNA were fused into GAL4 BD vectors, respectively. Then, the resulting construct of *OsDRB2* was co-transformed with the GAL4 BDs containing *DCL1-RBD* and *OsDRB1-2* into Y2H Gold yeast cells, respectively. The result showed that OsDRB2 protein can interact with OsDCL1 (DCL1-RBD) and OsDRB1-2 proteins, respectively (Figure 7B).

### 2.7. Expression Analyses of miRNAs and Their Target Genes Associated with Leaf Development

**Figure 8:**
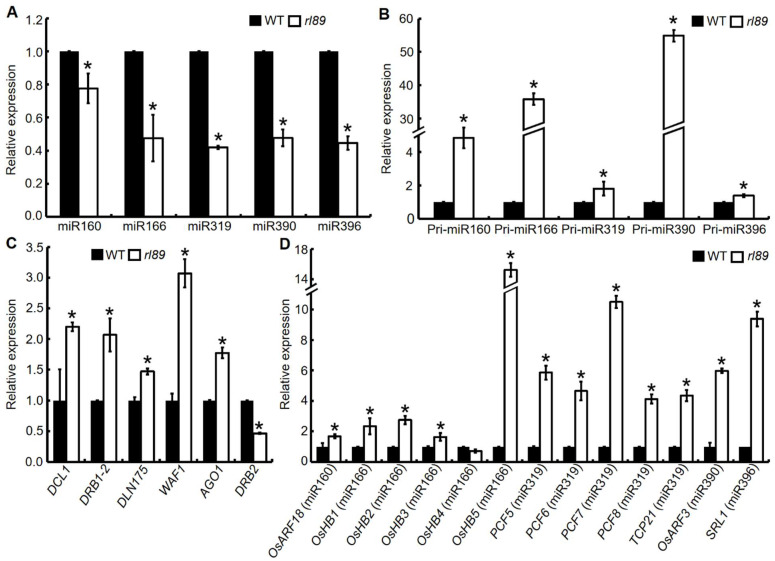
Expression analyses of miRNAs and their target genes related to leaf polarity and/or morphology development. (**A**) Relative expression of five miRNAs associated with leaf development. The 12th leaves (64 d after sowing, the leaf blades were just fully expanded) were harvested from the *rl89* mutant and its wild type (WT) for a stem-loop primer-based quantitative reverse transcription PCR (SL-qPCR). The relative mRNA amount of each miRNA was normalized to snRNA U6. The relative expression of each miRNA in WT was normalized to 1, and those in *rl89* were calculated accordingly. (**B**–**D**) Expression levels of the corresponding Pri-miRNAs, some genes encoding miRNA processing proteins, and some target genes of the five miRNAs, respectively. The total RNA was extracted from the 12th leaves (64 d after sowing) of *rl89* and WT, respectively. The relative mRNA amount of each gene in (**B**–**D**) was normalized to *Osubiquitin* and *OsActin* genes, respectively. The relative expression of each gene in WT was normalized to 1, and those in *rl89* were calculated accordingly. Error bars represent the SDs of three independent experiments. Asterisks indicate statistically significant differences compared with the wild type at *p* < 0.05.

In Arabidopsis, a loss of DRB2 activity obviously affected expression levels of some miRNAs and Pri-miRNAs [21,28]. In rice, some miRNAs were shown to be involved in the regulation of leaf polarity and/or morphology development, such as miR160, miR166, miR319, miR390, and miR396 [34,35]. So, we detected the expression changes of these miRNAs and their primary miRNAs in the 12th leaves of *rl89* mutant because *rl89* started to show leaf rolling when the 12th leaves emerged (Figure 1A–C). The result showed that miR160, miR166, miR319, miR390, and miR396 were obviously down-regulated (Figure 8A), whereas their primary miRNAs (Pri-miR160, Pri-mi166, Pri-mi319, Pri-mi390, and Pri-mi396) were all significantly accumulated in the mutant (Figure 8B). Given the obvious changes in miRNAs expression in this mutant, we also examined expression levels of genes associated with miRNA biogenesis in the 12th leaves of *rl89* and its wild type, and the result showed that *DCL1*, *DRB1-2*, *DLN175* (*DLN REPRESSOR 175*), *WAF1* (*WAVY LEAF1*), and *AGO1* had significantly up-regulated expressions in the mutant (Figure 8C). In addition, because miR162 and miR168 target *DCL1* and *AGO1,* respectively [21,28], we further analyzed expression changes of the two miRNAs, showing that their expressions were unchanged in *rl89* (Figure S7).

miRNAs can regulate gene expression by binding to mRNA complementary sequences for transcript destabilization [36]. In rice, it has been reported or predicted that *AUXIN RESPONSIVE FACTOR* gene (*OsARF18*), five HD-Zip III genes (*OsHB1* to *OsHB5*), five TCP transcription factor family genes (*OsPCF5* to *OsPCF8*, and *OsTCP21*), *OsARF3* gene and *SRL1* gene were targeted by miR160 [34], miR166 [35,37,38], miR319 [35], miR390 [35], and miR396 (http://www.pmiren.com/, accessed around 11 October 2019), respectively, and transgenic plants overexpressing some of these target genes showed rolled leaf phenotype. Therefore, we further investigated expression levels of the miRNA target genes in the 12th leaves of *rl89*. As shown in Figure 8D, except for *OsHB4*, the other 12 target genes exhibited significantly increased expression in the *rl89* leaves, compared with those in the wild-type leaves.

Furthermore, we examined expressions of these miRNAs and their target genes in flag leaves of the *rl89* mutant and the *OsDRB2* KO lines, respectively. As shown in Figure S8, the five miRNAs were all dramatically down-regulated, and the 13 target genes were almost all significantly up-regulated in *rl89* mutant and the KO lines, which were basically consistent with those in the 12th leaves of *rl89*. For comparison, we also examined expressions of these miRNAs and target genes in the fourth leaves (at seedling stage) and the eighth leaves (at tillering stage) of *rl89* when its leaves were normal (unrolled), showing that these miRNAs and target genes were almost unchanged in this mutant (Figure S9).

Taken together, the above data indicated that the mutated *OsDRB2* gene could lead to the rolled leaf phenotype of *rl89* via multiple regulatory pathways consisting of miRNA biogenesis-related genes, miRNAs, and their target genes for leaf polarity and/or morphology development.

### 2.8. Expression Analyses of Some Genes Regulating Leaf Shape and Cell Growth

**Figure 9:**
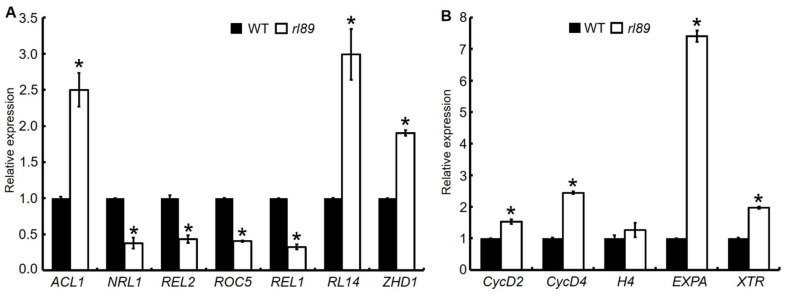
Expression analyses of some genes associated with leaf shape and cell growth. Comparison of expression levels of leaf shape-related genes (**A**) and cell growth-related genes (**B**) were performed between *rl89* and the wild type (WT). The total RNA was extracted from the 12th leaves (64 d after sowing, the leaf blades were just fully expanded) of *rl89* and WT, respectively. The relative mRNA amount of each gene was normalized to *OsActin*. The relative expression of each gene in WT was set to 1.0, and those in *rl89* were calculated accordingly. Error bars represent the SDs of three independent experiments. Asterisks indicate statistically significant differences compared with the wild type at *p* < 0.05.

The rolled leaf phenotype of *rl89* is resulted from the reduced number and size of bulliform cells in its leaves. Correspondingly, it was reported that some rice genes, such as *ACL1*, *NRL1*, *REL2*, *ROC5*, *REL1*, *RL14,* and *ZHD1*, play important roles in regulating development of bulliform cells involving in leaf shape [3,8,9,11,12,13,16]. So, we analyzed expression levels of the seven genes above-mentioned in the 12th leaves of *rl89* mutant and its wild type. As a result, *ACL1*, *RL14,* and *ZHD1* expressions were significantly up-regulated, and the other four genes (*NRL1*, *REL2*, *ROC5,* and *REL1*) showed significantly down-regulated expressions in the *rl89* mutant, compared with those in the wild type (Figure 9A).

Meanwhile, we examined transcription levels of the five genes for cell cycle and cell elongation in the 12th leaves. Among them, Cyclin D2 (*CycD2*), Cyclin D4 (*CycD4*), and histone *H4* genes are mainly related to cell cycle, whereas α-Expansin (*EXPA*) gene and xyloglucan endotransglycosylase-related (*XTR*) gene are associated with cell elongation [39]. As shown in Figure 9B, except *OsH4*, expressions of *OsCycD2*, *OsCycD4*, *OsEXPA,* and *OsXTR* genes were all significantly up-regulated in *rl89* mutant, compared to those in the wild type.

Furthermore, we investigated expression levels of the above 12 genes in flag leaves of the *rl89* mutant and the *OsDRB2* KO lines, respectively. As a result, seven genes (*ACL1*, *RL14*, *ZHD1*, *OsCycD2*, *OsCycD4*, *OsEXPA,* and *OsXTR*) were significantly up-regulated, four genes (*NRL1*, *REL2*, *ROC5,* and *REL1*) were significantly down-regulated, and only one gene (*OsH4*) was unchanged in flag leaves of both *rl89* and KO (Figure S10), showing that the expression changes of the 12 genes in the flag leaves were basically consistent with those in the 12th leaves of *rl89* mutant. By contrast, we also examined expressions of the 12 genes in the fourth leaves and the eighth leaves of *rl89*, showing that these genes were almost unchanged in this mutant (Figure S11).

To sum up, these results suggested that mutation of *OsDRB2* also affected expression of some genes regulating leaf shape and cell growth.

## 3. Discussion

So far, *DRB2* genes have been characterized from dicotyledonous plants such as Arabidopsis and soybean, using T-DNA insertion mutant or CRISPR/Cas9 technique [28,29]. However, no *DRB2* gene has been identified in monocotyledonous plants. In this study, a rice leaf rolling mutant *rl89* was isolated, which showed adaxially rolled leaf phenotype due to decreased number and size of bulliform cells on the adaxial side of leaves (Figure 1B–D and Figure 2A–F,P,Q). Then, we cloned the *OsDRB2* gene by means of the *rl89* mutant and MutMap cloning approach. In this mutant, a C to T substitution in the *OsDRB2* gene resulted in an amino acid change in the encoded protein (Figure 3D). Further, the leaf rolling phenotype of *rl89* could be rescued by introducing the wild-type *OsDRB2* gene (Figure 2G–I,P,Q and Figure 4A,B,E,F). Moreover, the CRISPR/Cas9-mediated *OsDRB2* knockout mutants also exhibited adaxially rolled leaves due to reduced number and size of bulliform cells (Figure 2J–Q and Figure 4C–F). Therefore, we confirmed that the point mutation of *OsDRB2* was the cause of the leaf rolling phenotype of *rl89*, and successfully identified an *OsDRB2* gene involved in miRNA biogenesis in rice.

In plants, most miRNAs modulate transcript levels of target genes by transcript cleavage, which plays an important role in guiding the development of leaf polarity [40,41]. In Arabidopsis, miR165/166 negatively regulated HD-Zip III genes *PHABULOSA* (*PHB*), *PHAVOLUTA* (*PHV*), and *REVOLUTA* (*REV*), and then regulated adaxial/abaxial patterning of leaves [42]. In maize, the expression of HD-Zip III family member *ROLLED LEAF1* (*Rld1*) was also regulated spatially through miR166, which determined adaxial/abaxial polarity in developing leaves [43]. In rice, overexpressions of the miR166-resistant versions of HD-Zip III genes *OsHB1* (*OsHB1m*) and *OsHB3* (*OsHB3m*) caused adaxially rolled leaves and radialized filamentous leaves, respectively, indicating that some HD-Zip III genes are important regulators in development of the adaxial/abaxial patterning of leaves [37]. Similarly, the knockdown lines of miR166 showed the increased expression of four *OsHB* genes, leading to rolled leaves due to smaller bulliform cells. Meanwhile, *rOsHB4* overexpression lines also exhibited leaf rolling [38]. Recent study reported that a point mutation in *LF1* (*OsHB1* allele), which was just located in a putative miRNA165/166 target sequence, caused adaxially rolled leaf due to the ectopic formation of bulliform-like cells on the abaxial side of leaves [44]. In this study, the *rl89* mutant and *OsDRB2* KO lines displayed adaxially rolled leaves due to decreased number and size of bulliform cells on the adaxial side of leaves. However, the adaxial/abaxial polarity in leaves of *rl89* mutants and *OsDRB2* KO lines looked normal. At the same time, qRT-PCR showed that five miRNAs (miR160, miR166, miR319, miR390, and miR396) involved in leaf polarity and/or morphology had significantly down-regulated expressions, whereas almost all of their target genes had significantly up-regulated expressions in the leaves of *rl89* and *OsDRB2* KO lines (Figure 8A,D and Figure S8). Taken together, defects of the *OsDRB2*-miR166-*OsHBs* pathway could play an important role in formation of the rolled leaf phenotype of *rl89*. In this pathway, the *OsDRB2* mutation in *rl89* reduced miR166 accumulation, then elevated expressions of HD-Zip III genes (such as *OsHB1*, *3,* and *5*), and finally caused leaf rolling, which is consistent with conserved function of miR166 and HD-Zip III genes in establishment of leaf polarity and morphology. Moreover, the *OsDRB2* mutation also reduced accumulation of miR160, miR319, miR390, and miR396 in *rl89*, which could also cause the abnormal leaf development by regulating the expression of their target genes related to leaf development.

Both number and size of bulliform cells were obviously decreased in *rl89* leaves. It was reported that *NRL1* and *REL2* are involved in regulating the adaxial bulliform cell development of leaves through different pathways in rice, and down-regulation of *NRL1* and *REL2* expression resulted in the defects of adaxial bulliform cells [3,16]. Meanwhile, *ACL1*, *ROC5*, *REL1*, *RL14,* and *ZHD1* genes were also reported to affect number and size of bulliform cells in rice leaves [8,9,11,12,13]. In this study, expression levels of *NRL1*, *REL2*, *ROC5,* and *REL1* genes were significantly down-regulated, and those of *ACL1*, *RL14,* and *ZHD1* genes were significantly up-regulated in *rl89* mutant and *OsDRB2* KO lines (Figure 9A and Figure S10A,C). In addition, rice plants overexpressing *OsYABBY6* displayed abnormally development of bulliform cells in the adaxial surface, which might be related to increased expression of the five genes related to cell growth (*OsCycD2*, *OsCycD4*, *OsH4*, *OsEXPA,* and *OsXTR*) [39]. In *rl89* and KO lines, except *OsH4*, expressions of the other four genes were significantly up-regulated (Figure 9B and Figure S10B,D). Collectively, expressions of some genes related to leaf shape and cell development were significantly altered, which implied that some unexplored miRNAs might target these genes and then regulate their accumulation, finally affecting the development of bulliform cells in *rl89* leaves.

Arabidopsis T-DNA insertion *drb2* mutant was normal at the seedling stage [45], and showed rosette leaves with margin serration, ovoid, flatter, and darker in color at four weeks after sowing [28]. Soybean CRISPR/Cas9 knockout *drb2ab* double mutant displayed dark green leaf coloration [29]. In this study, both the *rl89* mutant resulted from a single-base mutation of *OsDRB2* and the CRISPR/Cas9 knockout *drb2* mutant were normal at the early stage of growth but showed leaf-rolling phenotype at the late stage of growth (Figure 1A–D and Figure 4C–F). This phenotype caused by the *OsDRB2* mutation was obviously different from those of the Arabidopsis and soybean *drb2* mutants. In aspect of gene expressions, the expression level of *DRB2* in Arabidopsis *drb2* leaves was lower throughout development [21]. In this study, the expression level of *OsDRB2* was lower at seedling stage but remarkably increased at booting stage (Figure 6A). Accordingly, some miRNAs and related genes in *rl89* leaves were almost unchanged at the early stage of growth but obviously up-regulated at the late stage of growth (Figure 8A,D, Figure 9 and Figures S8–S11), which were consistent with the change of leaf rolling phenotype of *rl89*. On the other hand, microarray assay suggested that transcription levels of almost all target genes of miRNAs (including *ATHB14* and *PHV* homologous to *OsHB3* and *OsHB4* respectively) of miRNAs in Arabidopsis *drb2* plants were similar to those in wild-type plants. Nonetheless, the proteome analyses for 10 miRNA targets showed that the elevated protein accumulation in the *drb2* background were relative to those in wild-type plants. These data strongly suggested that DRB2 is required for translation inhibition and that this process is inoperative in *drb2* [21]. However, our qRT-PCR analysis showed that the transcription levels of almost all target genes of miRNAs in *rl89* mutant were significantly up-regulated (Figure 8D and Figure S8B), implying that *OsDRB2* could regulate transcriptions of miRNA target genes in different way(s) to Arabidopsis *DRB2*. The above discrepancies of phenotypes and gene expressions could be due to functional divergence of *DRB2*, some miRNAs and/or related genes between monocotyledonous and dicotyledonous plants or in different plant species.

qRT-PCR analysis showed that five miRNAs (miR160, miR166, miR319, miR390, and miR396) were obviously down-regulated (Figure 8A), whereas their primary miRNAs (Pri-miRNAs) were all significantly accumulated in the *rl89* mutant (Figure 8B), suggesting that *OsDRB2* is involved in the processing of some Pri-miRNA transcripts. On the other hand, in this mutant, five genes (*DCL1*, *DRB1-2*, *DLN175*, *WAF1,* and *AGO1*) involved in miRNA biogenesis had significantly up-regulated expressions (Figure 8C). But expressions of miR162 and miR168 targeting *DCL1* and *AGO1*, respectively, were unchanged (Figure S7), implying that the elevated expression of the miRNA-biogenesis genes should not be resulted from expression changes of the miRNAs targeting these genes for miRNA biogenesis. Taken together, when OsDRB2 function was weakened in *rl89*, elevated expressions of the five genes for miRNA biogenesis in *rl89* might result from feedback regulatory network among these miRNA-biogenesis genes. This feedback regulation might partially compensate for reduced accumulation of the five miRNAs, which were resulted from the *OsDRB2* mutation.

## 4. Materials and Methods

### 4.1. Plant Materials

Rice *rolled* *leaf* mutant *rl89* was obtained from the mutagenized population of *indica* rice restorer line Lehui188 (188R) by ethyl methanesulfonate (EMS). The mutant was crossed with the wild type 188R to construct the F_1_ and F_2_ populations for genetic analysis, and it was also crossed with *japonica* rice variety Nipponbare to construct the mapping population of the *rl89* locus. Rice materials were planted in a paddy field under the local rice growing season in Wenjiang District (latitude 30°42′ N, longitude 103°50′ E and altitude 539.3 m), Chengdu, Sichuan, China.

### 4.2. Determination of Photosynthetic Parameter and Leaf-rolling Index (LRI)

Net photosynthetic rate, stomatal conductance, and transpiration rate of the flag leaves of *rl89* mutant and its wild type at the heading stage were measured by portable photosynthetic apparatus (Li-6400, Li-COR Inc., Lincoln, NE, USA). All measurements were conducted under 400 ppm of CO_2_ concentration, sunny weather condition and the solar radiation of approximately 1200 μmol m^−2^ s^−1^, during 09:30–10:30 a.m. [46]. On the other hand, widths of the top three leaves of rice plants were measured under natural (Ln) and unfolding (Lw) states, respectively, and LRI values were calculated as LRI (%) = ((Lw − Ln) ÷ Lw) × 100 [47].

### 4.3. Measurement of Photosynthetic Pigments

Leaf samples were collected from *rl89* and its wild type at heading stage. In total, 0.2 g fresh leaves were used to extract photosynthetic pigments with 80% acetone at 4 °C for 48 h in the dark. Then, contents of chlorophylls (Chl) and carotenoids (Caro) were measured using a BIOMATE 35 UV-Visible Spectrophotometer (Thermo Scientific, Waltham, MA, USA) at 470 nm, 646 nm, and 663 nm, and were calculated according to the method of Lichtenthaler and Wellburn [48]. The pigment data of each sample were measured with three technical replicates for each of three independent biological experiment repeats.

### 4.4. Histology Analysis

For paraffin section analysis, the basal half of each flag leaf of rice plants was collected and fixed in FAA solution (70% ethanol, 38% formaldehyde, glacial acetic acid, and glycerol) for 24 h, and treated with a series ethanol (75, 85, 90, 95, and 100%). Then, the samples were infiltrated and embedded in paraffin. Subsequently, sections (approximately 5–10 μm thick) were cut with a microtome (Leica RM2016), stained with 1% (*w*/*v*) safranin O and 1% (*w*/*v*) fast green FCF (G1031), examined with a fluorescence microscope (eclipse 80I; Nikon, Tokyo, Japan), and photographed. Finally, number of bulliform cell in the midrib, large veins, and small veins of the flag leaf were counted, and their areas were measured with Image J software.

### 4.5. MutMap Analysis and Marker Development

To identity candidate gene of the *rl89* locus, we isolated DNA of 30 individuals showing the mutant phenotype in the (*rl89* × 188R) F_2_ progenies and bulked the samples in an equal ratio. This bulked DNA was subjected to whole-genome sequencing. Subsequently, MutMap analysis was performed to detect single nucleotide polymorphisms (SNPs) between genomes of *rl89* mutant and 188R using Nipponbare genome as the reference genomic sequence [49]. The genome of 188R was re-sequenced at the same time and used as a control. Whole-genome sequencing and MutMap analysis were conducted by Novogene Biotech Co., Ltd. (Beijing, China). For verification of MutMap result, insertion/deletion (InDel) markers were designed around SNPs linked genetically to the *rl89* locus using the Primer 5.0 software, and the genetic linkage map of *rl89* was constructed by using the InDel markers and the mutant plants from the (*rl89* × *japonica* cv. Nipponbare) F_2_ population. The DNA of each sample was extracted from rice leaves with cetyltrimethylammonium bromide (CTAB) or sodium dodecyl sulfonate (SDS) methods.

### 4.6. Sequence Analysis

The amino acid sequences of OsDRB2 and its homologues were acquired from Gene Bank (http://www.ncbi.nlm.nih.gov, accessed around 20 December 2021). Multiple sequence alignment was conducted using DNAMAN version 9.0 (Lynnon Biosoft, Foster City, CA, USA). The phylogenetic tree was constructed using the program MEGA 7.0 (Mega Limited, Auckland, New Zealand) and the maximum likelihood algorithm.

### 4.7. Vector Construction and Rice Transformation

To construct complementation vector of the *rl89* mutant, the promoter and genome sequence of *OsDRB2* (6191 bp) gene was amplified from the genome of wild-type 188R by PCR using the primers PF1: 5′-GAGCTCGGTACCCGGGGATCCGTAGCAACCTCTAGCATCTGTA-3′ and PR1: 5′-CAGGTCGACTCTAGAGGATCCTGGGTCAGCACACTGTAG-3′ (both PF1 and PR1 contained *Bam*HI site), and the PCR product was ligated into the binary vector pCAMBIA1300 by *Bam*HI digestion, generating the pCAMBIA1300-*OsDRB2* plasmid. Meanwhile, the CRISPR/Cas9 knockout vector of *OsDRB2* was also constructed as previously described method [30]. Subsequently, the complementation and knockout vectors were transformed into the *rl89* mutant and the *japonica* rice variety Zhonghua 11 (ZH11) by *Agrobacterium* strain EHA105-mediated transformation, respectively. The complementary transgenic positive plants were examined by using the primers 3970-10F: 5′-TGGGACCGCTGGTTGTGG-3′ and pC13-151R: 5′-GGGCCTCTTCGCTATTAC-3′, which were located on the *OsDRB2* gene and the pCAMBIA1300 vector, respectively. The knockout positive transgenic plants were detected using primers SG9932F: 5′-GTTACTTCTGTTTCAATC-3′ and SG9932R: 5′-CAATGGTACAGTGGAACG-3′.

### 4.8. Histochemical GUS Assay

To investigate the tissue expression, a 2234 kb promoter sequence (upstream from start codon ATG) of the *OsDRB2* was amplified with specific primers (the upper primer 5′-TGGCTGCAGGTCGACGGATCCGTAGCAACCTCTAGCATCTGTA-3′ and lower primer 5′-CCAGTGAATTCCCGGGGATCCCGCCGCCGCCGCCGCAGC-3′), and then fused into pCAMBIA1391Z after digestion with *Bam*HI. The resulting *OsDRB2*_Pro_::GUS construct was transformed into the ZH11 by *Agrobacterium*-mediated method. The positive transgenic plants were examined using the primers GUSF: 5′-GCTCACTCATTAGGCACC-3′ (on pCAMBIA1391Z vector) and 3970-R2: 5′-ATGCTTTGCGGTTACTAG-3′ (on *OsDRB2* gene promoter). The GUS activities of organs and tissues at the seedling and booting stages were detected by the instruction of GUS staining kit (Coolaber).

### 4.9. Subcellular Localization

To investigate the subcellular localization of OsDRB2 protein, the 1545-bp of cDNA fragment of *OsDRB2* was amplified from the wild-type 188R using the primers gc970F: 5′-CGGGGATCCTCTAGAGTCGACATGTATAAGAACCAGCTC-3′ and gc970R: 5′-CACCATGGTACTAGTGTCGACTGATTTGAGCTCGAGATG-3′, which contained *Bam*HI site at the 5′-end and the 3′-end of the cDNA fragment. The constructed fusion vector pCAMBIA2300-35S-*OsDRB2*-eGFP and the empty vector pCAMBIA2300-35S-eGFP (as a control) were co-transformed with *WRKY45*-RFP (as nuclear marker), respectively, into the *Nicotiana benthamiana* leaves, following the method as previously described [50]. GFP fluorescence in the *N. benthamiana* leaves was observed using a laser scanning confocal microscope (Nikon A1, Nikon, Japan).

### 4.10. Yeast-Two-Hybrid (Y2H) Assay

The Matchmaker GAL4 two-hybrid system 3 of Clontech (Clontech, Mountain View, CA, USA) was used. The cDNA sequences of *OsDRB2*, *OsDCL1-RBD* domain and *OsDRB1-2* were amplified and inserted into pGADT7 and pGBKT7 vectors, respectively, and the *Bam*HI site in the vectors was chosen as the insertion site for all sequences. The interaction of pGADT7-T with pGBKT7-Lam and the interaction of pGADT7-T with pGBKT7-53 were used as negative and positive controls, respectively. The self-activation was tested by the bait construct fused with BD. The above recombinant vectors were combined according to experimental needs, and then co-transformed into the yeast strain Y2HGold cells. After that, each transformant was spotted on a non-selective medium (−Leu/−Trp) plates for about 3 d at 28 °C and tested for the protein interaction by observing their growth situation on selective (−Ade/−His/−Leu/−Trp) plates. Primer sets used for generating the above constructs were listed in Table S4.

### 4.11. qRT-PCR Analyses

The different tissues of rice were sampled from 7 to 8 a.m. Total rice RNA was extracted with an RNA isolater kit (Vazyme), and the first-strand cDNA was reverse transcribed from total RNA (2 μg) using a reverse transcription kit (Vazyme). In order to enrich leaf miRNAs, the precipitation by isopropanol was carried out for 2 h at −20 °C and centrifuged. Then, the RNA pellets were further washed with 70% ethanol and air-dried [33], and miRNA first-strand cDNA synthesis kit (by stem-loop, Vazyme) was selected to elongate and reverse transcribe miRNA following the manufacturer′s instructions.

In order to analyze expression differences of genes between the mutant and its wild type, 43 genes were detected by qRT-PCR, including seven miRNAs, five Pri-miRNAs, six miRNA processing-related genes, thirteen target genes of miRNAs, seven leaf shape-related genes, and five cell growth-related genes. The amplification of each gene was carried out in a total volume of 10 µL containing 0.1 µM of each primer and 1 × SYBR green PCR master mix (Vazyme) using the CFX96 real-time PCR system (Bio-Rad). The program used in these qRT-PCR assays was as follows: 95 °C for 3 min, then 40 cycles of 95 °C for 10 s and 58 °C for 30 s. For each sample, qRT-PCR was performed with three technical replicates for each of three biological replicates, and snRNA U6 or *Osubiquitin* (*UBQ*) or *OsActin* was used for normalization as an internal control. The 2^−ΔΔCT^ method was applied to calculate relative changes in gene expression. All primers for qRT-PCR were listed in Tables S5–S7.

### 4.12. Statistical Analysis

The statistical analysis was performed by using Excel in Office 2016. All experiments were repeated independently for three replicates, and the data were subjected to statistical analysis using the Student’s *t*-test with a *p*-value less than 0.05 or 0.01 considered significant.

## Data Availability

The data presented in this study are available in the article or Supplementary Materials.

## Supplementary Materials

The supporting information can be downloaded at: https://www.mdpi.com/article/10.3390/ijms231911147/s1.
