# Supplementary material for: DRB2 Modulates Leaf Rolling by Regulating Accumulation of MicroRNAs Related to Leaf Development in Rice
Source: Int J Mol Sci. 2022 Sep 22;23(19):11147. doi: 10.3390/ijms231911147 (PMC9570175; doi:10.3390/ijms231911147)
Supplement: Supplementary file 1 [file ijms-23-11147-s001.zip › ijms-1906774-supplementary.pdf]

Supplementary Materials

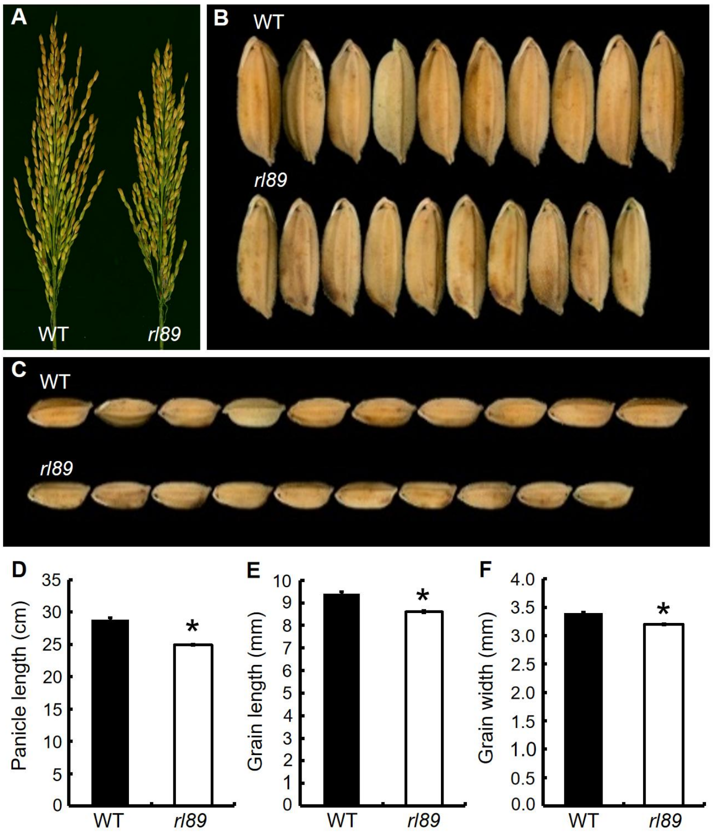

Figure S1. Comparison of panicles and grains between the *rl89* mutant and its wild-type 188R (WT). (**A**), (**B**) and (**C**) Comparison of panicle size, grain width and grain length, respectively. **(D)**, (**E**) and (**F**) Statistical analysis of panicle length, grain length and grain width, respectively. Error bars represent the standard deviations (SDs) of three independent experiments. * indicates statistically significant difference compared to the wild type at $P < 0.05$.

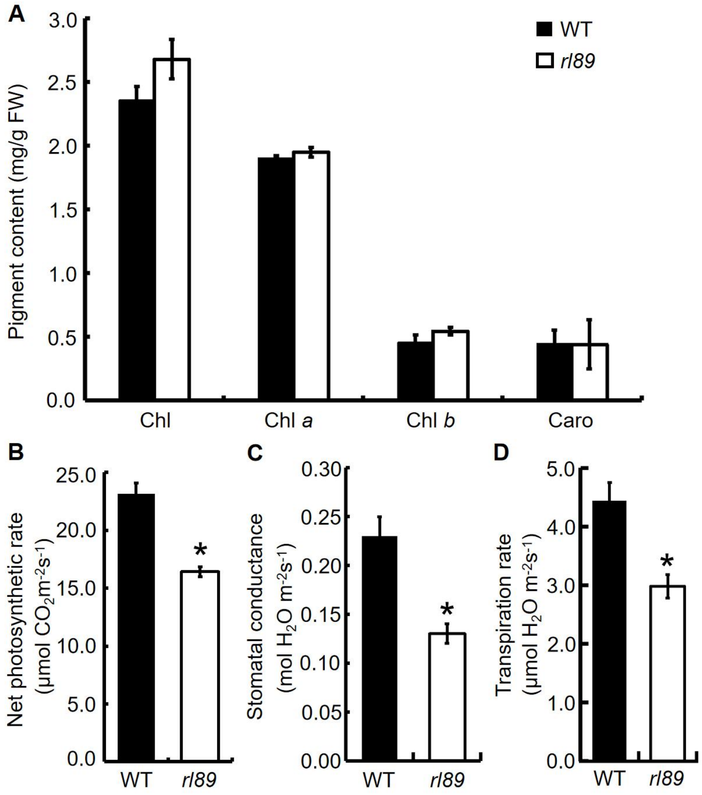

Figure S2. Pigment contents and photosynthetic parameters in flag leaves of the *rl89* mutant and its wild type (WT) at the heading stage. (**A**) Pigment contents. (**B**) Net photosynthetic rate. (**C**) Stomatal conductance. (**D**) Transpiration rate. Data are means ± SD (n = 3). Error bars represent standard deviations (SDs) of three independent measurements. Asterisks indicate statistically significant differences compared with WT at $P < 0.05$. Chl: chlorophyll. Chl *a*: chlorophyll *a*. Chl *b*: chlorophyll *b*. Caro: carotenoids.

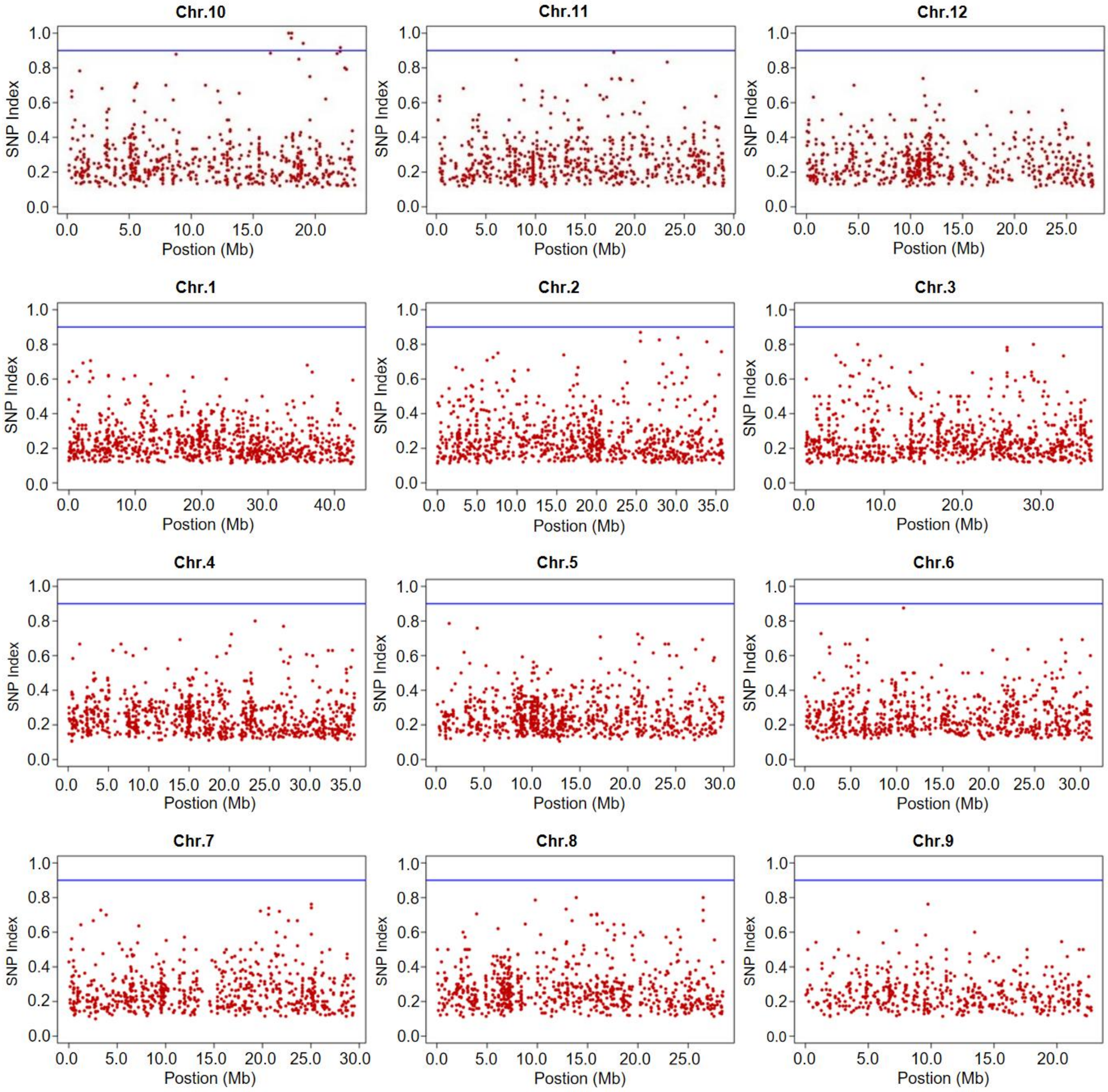

Figure S3. SNP index plots for 30 homozygous mutant plants from (*rl89*×188R) F_2 population.

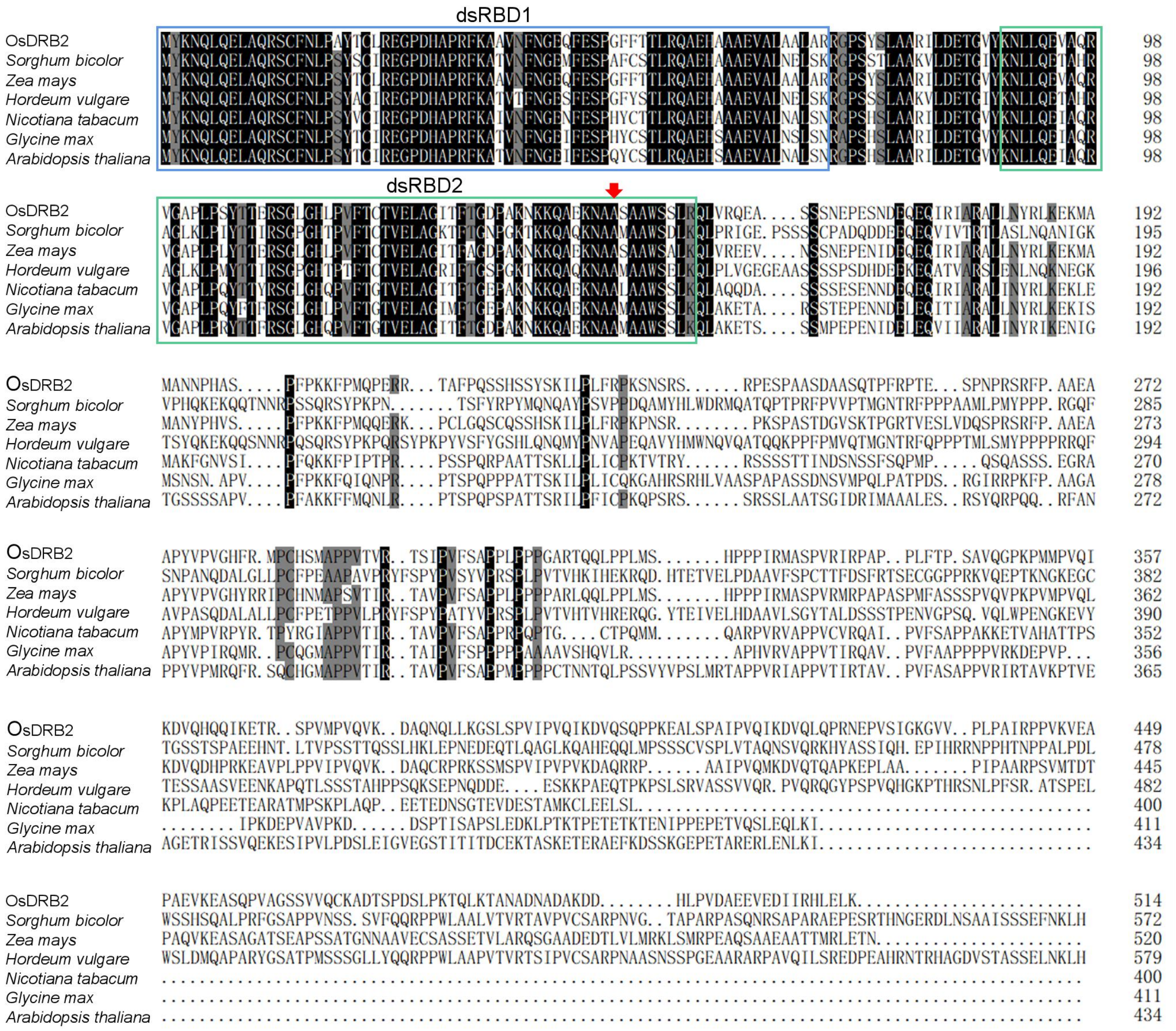

Figure S4. Sequence alignment of OsDRB2 and its homologues. Identical residues are shown on a black background, and similar residues (≥75% identical) are shown on a gray background. The blue box and the green box indicate the conserved dsRBD1 and dsRBD2 domains, respectively. The red arrow indicates the amino acid substitution from Ala-146 to Val in the *rl89* mutant. GenBank accession numbers for the respective protein sequences are as follow: OsDRB2 (*Oryza sative*, LOC_Os10g33970); SbDRB2 (*Sorghum bicolor*, XP_002440600.1); ZmDRB2 (*Zea mays*, ACG40426.1); HvDRB2 (*Hordeum vulgare*, KAE8779410.1); NtDRB2 (*Nicotiana tabacum*, XP_016505897.1); SbDRB2 (*Glycine max*, NP_001345291.1); and AtDRB2 (*Arabidopsis thaliana*, AT2G28380, NP_565672.1).

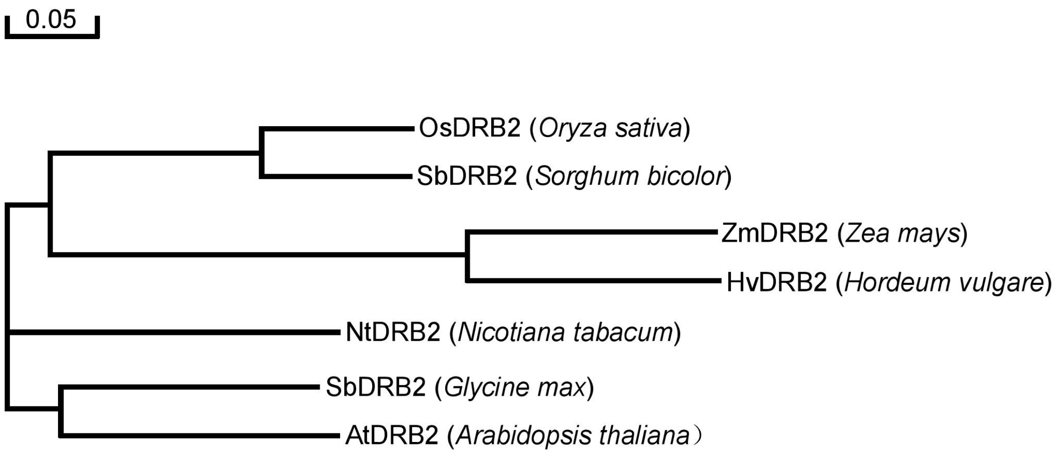

Figure S5. Phylogenetic analysis of OsDRB2 and its homologous proteins. The phylogenetic tree using percentage identities is based on a multiple sequence alignment generated with the program DNAMAN. The scale represents percentage substitution per site. GenBank accession numbers for the respective protein sequences are as Figure S4.

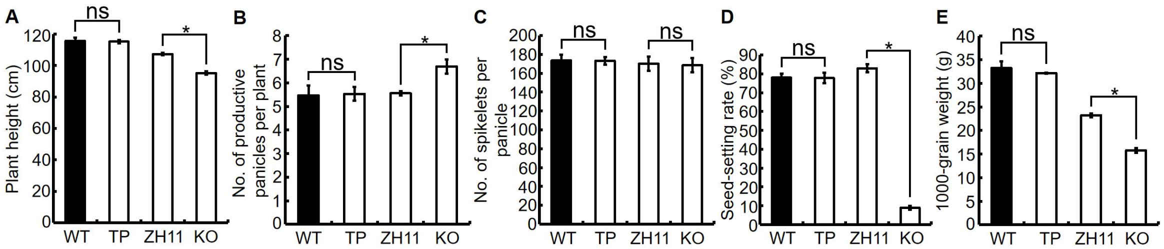

Figure S6. Major agronomic traits of the wild-type 188R (WT), the *OsDRB2* transgenic plants (TP), the control variety Zhonghua 11 (ZH11), and the *OsDRB2* knockout mutants (KO). (**A**) Plant height. (**B**) No. of productive panicles per plant. (**C**) No. of spikelets per panicle. (**D**) Seed-setting rate. (**E**) 1000-grain weight. Error bars represent the standard deviations (SDs) of three independent experiments. * and ns indicate statistically significant and non-significant differences respectively between WT and TP, or between ZH11 and KO at $P < 0.05$.

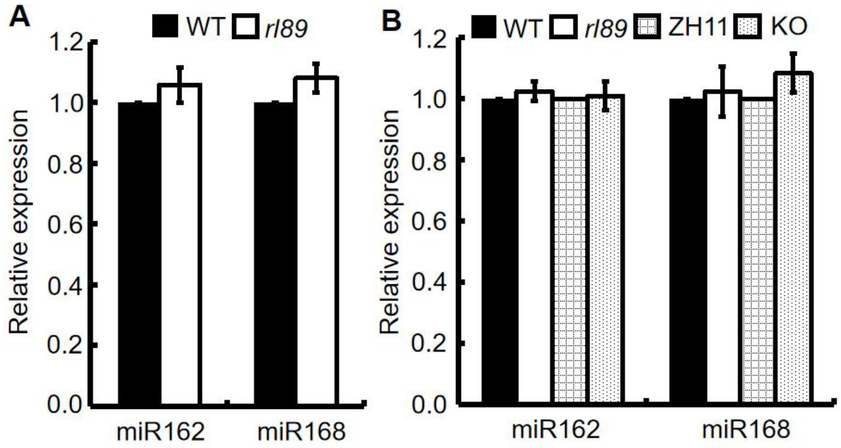

Figure S7. Expression analyses of miR162 and miR168 targeting *DCL1* and *AGO1*, respectively, in the *rl89* mutant and the *OsDRB2* knockout lines (KO). (**A**) The 12th leaves were harvested from *rl89* and WT for a stem-loop primer-based quantitative reverse transcription PCR (SL-qPCR). (**B**) The flag leaves were harvested from the *rl89* and its WT, and from the KO and the control variety Zhonghua 11 (ZH11), respectively, for a stem-loop primer-based quantitative reverse transcription PCR (SL-qPCR). The relative mRNA amount of each miRNA was normalized to snRNA U6. The relative expression of each miRNA in WT and ZH11 were set to 1.0, and those in *rl89* and KO were calculated accordingly. Error bars represent the SDs of three independent experiments. Asterisks indicate statistically significant differences compared with the wild type at $P < 0.05$.

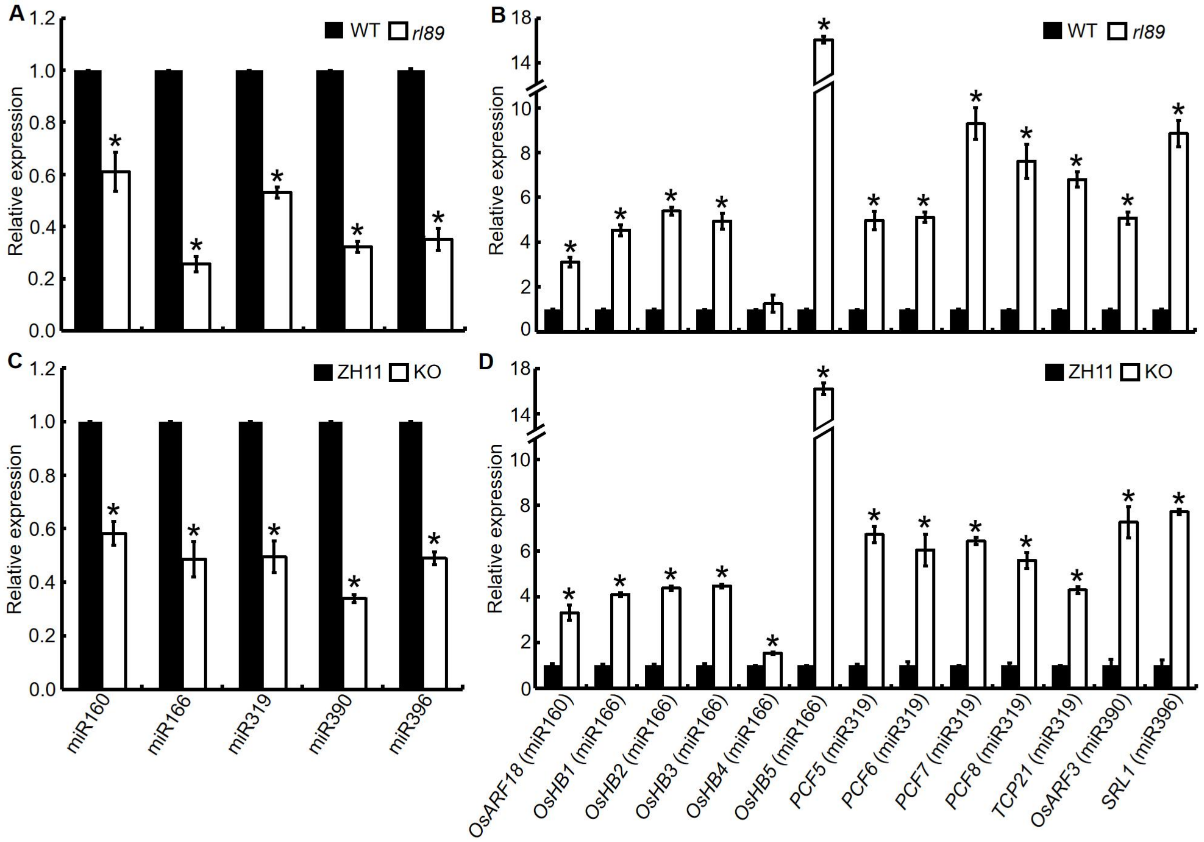

Figure S8. Expression analyses of miRNAs and their target genes related to leaf development in the flag leaves of the *rl89* mutant and the *OsDRB2* knockout lines (KO). (**A**) and (**C**) Relative expressions of five miRNAs in *rl89* and KO respectively. At the booting stage, the flag leaves were harvested from *rl89* and its wild type (WT), KO and the control variety Zhonghua 11 (ZH11) for a stem-loop primer-based quantitative reverse transcription PCR (SL-qPCR). The relative mRNA amount of each miRNA was normalized to snRNA U6. The relative expression of each miRNA was in WT and ZH11 normalized to 1, and those in *rl89* and KO were calculated accordingly. (**B**) and (**D**) Expression levels of some target genes of miRNAs in *rl89* and KO respectively. The total RNA was extracted from the flag leaves of *rl89*, WT, KO and ZH11 at the booting stage. The relative mRNA amount of each gene was normalized to *OsActin* genes. The relative expression of each gene in WT and ZH11 were set to 1, and those in *rl89* and KO were calculated accordingly. Error bars represent the SDs of three independent experiments. Asterisks indicate statistically significant differences compared with the wild type at $P < 0.05$.

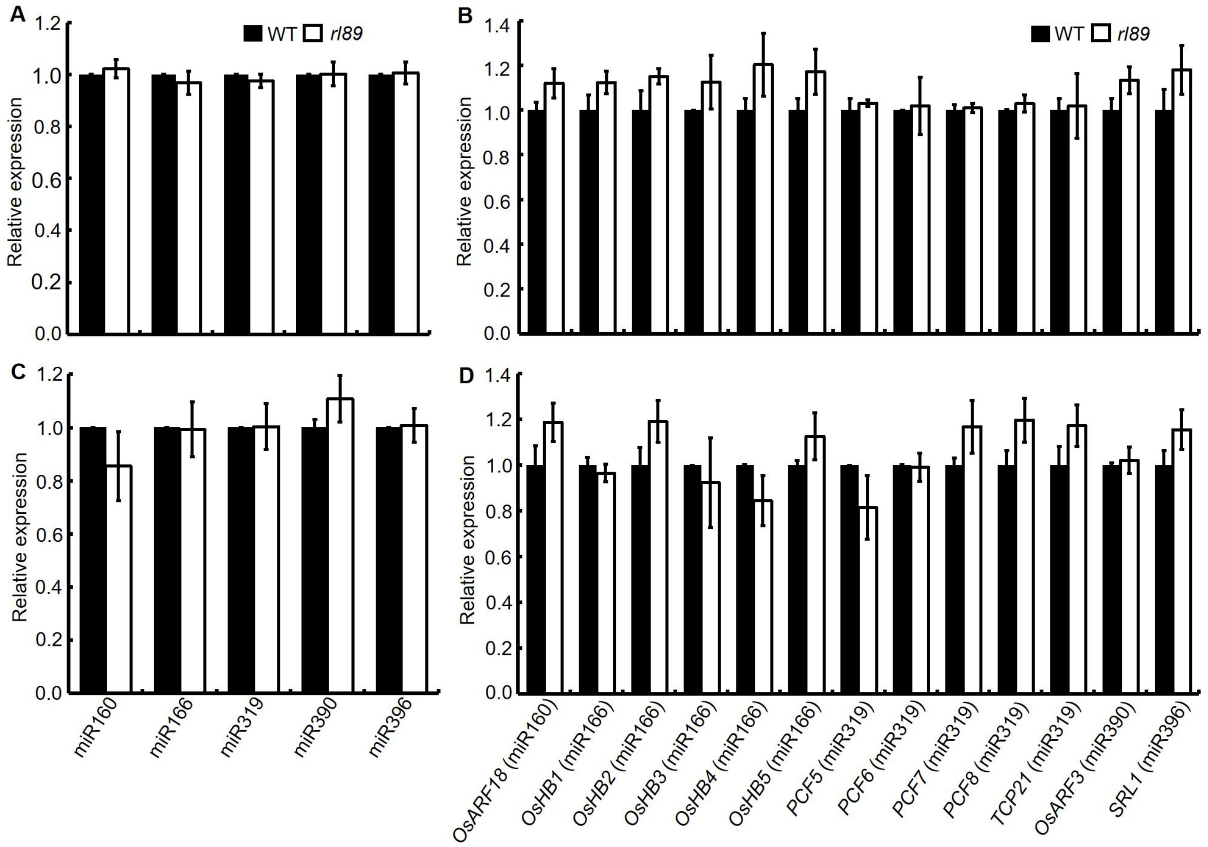

Figure S9. Expression analyses of miRNAs and their target genes related to leaf development in the fourth leaves (at seedling stage) and the eighth leaves (at tillering stage) of the *rl89* mutant. (**A**) and (**C**) Relative expressions of five miRNAs. The fourth leaves and the eighth leaves were harvested from the *rl89* mutant and its wild type (WT) for a stem-loop primer-based quantitative reverse transcription PCR (SL-qPCR). The relative mRNA amount of each miRNA was normalized to snRNA U6. The relative expression of each miRNA in WT was normalized to 1, and those in *rl89* were calculated accordingly. (**B**) and (**D**) Expression levels of some target genes of miRNAs. The total RNA was extracted from the fourth leaves and the eighth leaves of *rl89* and WT, respectively. The relative mRNA amount of each gene was normalized to *OsActin* genes. The relative expression of each gene in WT was normalized to 1, and those in *rl89* were calculated accordingly. Error bars represent the SDs of three independent experiments. Asterisks indicate statistically significant differences compared with the wild type at $P < 0.05$.

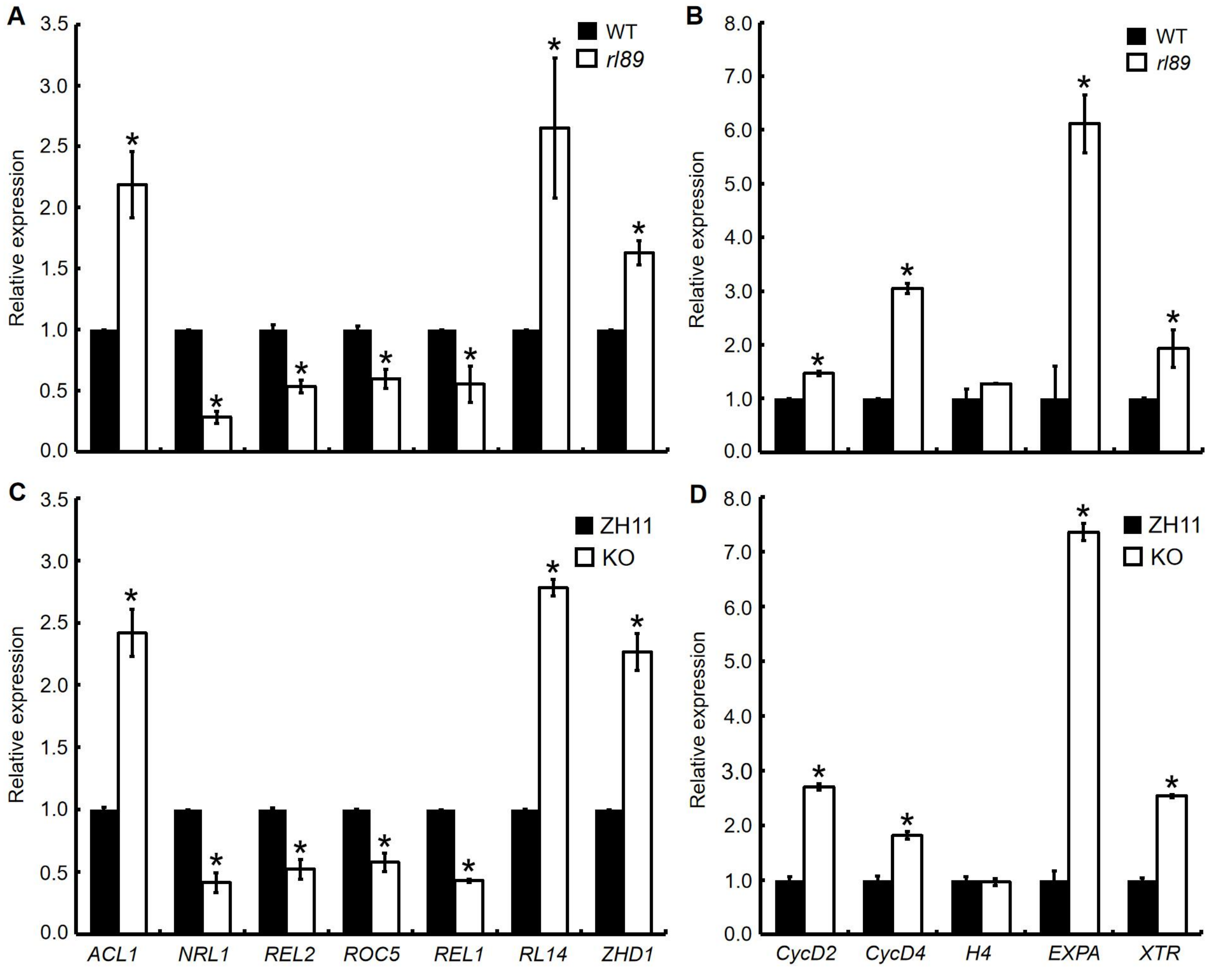

Figure S10. Expression analyses of some genes associated with leaf shape and cell growth in the flag leaves of *rl89* mutant and the *OsDRB2* knockout lines (KO). (**A**) and (**C**) Expression levels of leaf shape-related genes. (**B**) and (**D**) Expression levels of cell growth-related genes. The total RNA was extracted from the flag leaves of *rl89*, WT, KO and ZH11, respectively, at the booting stage. The relative mRNA amount of each gene was normalized to *OsActin*. The relative expression of each gene in WT and ZH11 were set to 1, and those in *rl89* and KO were calculated accordingly. Error bars represent the SDs of three independent experiments. Asterisks indicate statistically significant differences compared with the wild type at $P < 0.05$.

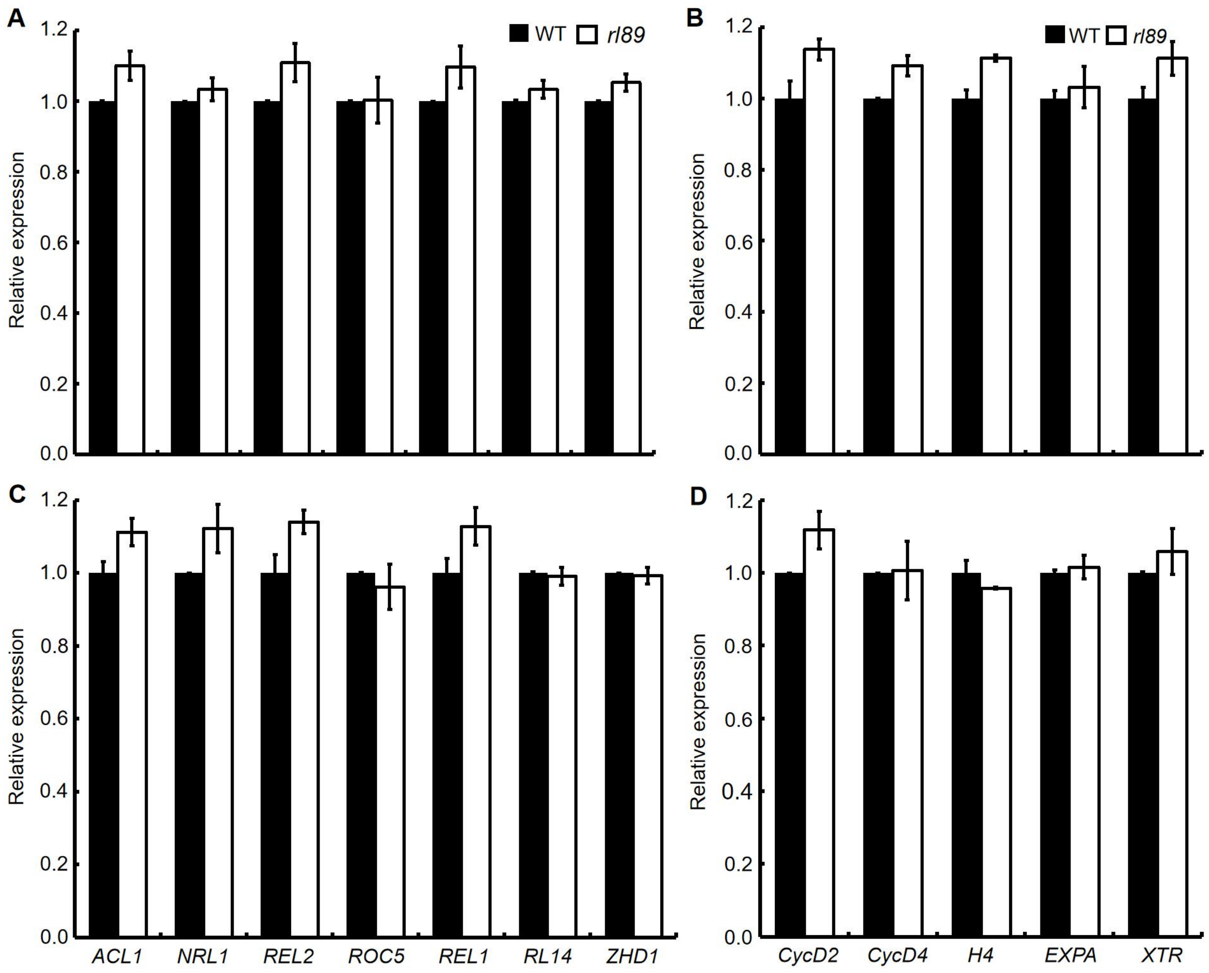

Figure S11. Expression analyses of some genes associated with leaf shape and cell growth in the fourth leaves (at seedling stage) and the eighth leaves (at tillering stage) of the *rl89* mutant and its wild type (WT). (**A**) and (**C**) Expression levels of leaf shape-related genes in the fourth leaves and the eighth leaves, respectively. (**B**) and (**D**) Expression levels of cell growth-related genes in the fourth leaves and the eighth leaves, respectively. The total RNA was extracted from the fourth leaves and the eighth leaves of *rl89* and WT, respectively. The relative mRNA amount of each gene was normalized to *OsActin*. The relative expression of each gene in WT was normalized to 1, and those in *rl89* were calculated accordingly. Error bars represent the SDs of three independent experiments. Asterisks indicate statistically significant differences compared with the wild type at $P < 0.05$.

Table S1. Segregation of rolling leaf trait in F_2 population of the cross between *rl89* and 188R.

Combination	No. of total plants	No. of flat leaf plants	No. of rolling leaf plants	Expected ratio	χ^2	$\chi^2_{0.05}$
rl89×188R	384	278	106	3:1	1.25	3.84

Table S2. Insertion/deletion (InDel) markers used for mapping of the *rl89* locus.

Maker	Forward primer (5′–3′)	Reverse primer (5′–3′)
p171	AGGCTGTCATCGCTTTCC	TAGGCTTTGTTCTATGCTTCC
p173	CTGACGAAGTACACCTCCTG	GTCCCAAACCTCTAAATGAA
p181	AGGTGGCTTGGTGTTGGT	TTCGCTGTGCTCTGGTTC
p183	CGGCTATGTATTTTACTCTT	CTATCTTCTTGGCTTTCG
p184	GGTAGCGGTCCTATCTTG	CCTCCATCTCCTCCTTCAC

Table S3. The primers for sequencing *LOC_Os10g33970.*

NO.	Name	Sequence
1	3970-F5	CGGGTGTGGAAATCGGTA
2	3970-F6	CTAGCTGACTATACATGG
3	3970-F7	GTACTGGTCCATGCTCCA
4	3970-F8	GATGCCTGTTCAGGTGAA
5	3970-F9	AATCCACATGCTTCACCC
6	3970-R2	ATGCTTTGCGGTTACTAG
7	3970-R3	GAAGAACCCGGGGCTCTCG
8	3970-R4	TACGTAATCAGGGTTTAG
9	3970-R5	TTCATGGCAGCAGTACTGG
10	3970-R6	ATATAATTCCTCCTGCTC
11	M13F (-47)	CGCCAGGGTTTTCCCAGTCACGAC
12	M13R (-48)	AGCGGATAACAATTTCACACAGGA

Table S4. Primers used in yeast two-hybrid.

Primer name	Sequence (5'-3')
DRB2-AD-F	GTGGGCATCGATACGGGATCCATATGTATAAGAACCAGCTC
DRB2-AD-R	CAGCTCGAGCTCGATGGATCCTTTGAGCTCGAGATGCCT
DRB2-BD-F	AGGCCGAATTCCCGGGGATCCGTATGTATAAGAACCAGCTC
DRB2-BD-R	CCGCTGCAGGTCGACGGATCCTTATTTGAGCTCGAGATG
DCL1-RBD-BD-F	AGGCCGAATTCCCGGGGATCCGTAGAGACCCTTCCGATGCA
DCL1-RBD-BD-R	CCGCTGCAGGTCGACGGATCCTCAGGGAAATCTCTATTC
DCL1-RBD-AD-F	GTGGGCATCGATACGGGATCCATAGAGACCCTTCCGATGCA
DCL1-RBD-AD-R	CAGCTCGAGCTCGATGGATCCTCAGGGAAATCTCTATTC
DRB1-2-BD-F	AGGCCGAATTCCCGGGGATCCGTATGGACATGCCGCCCACG
DRB1-2-BD-R	CCGCTGCAGGTCGACGGATCCTTATTCTTCGCTCATATT
DRB1-2-AD-F	GTGGGCATCGATACGGGATCCATATGGACATGCCGCCCACG
DRB1-2-AD-R	CAGCTCGAGCTCGATGGATCCTTCTTCGCTCATATTAGT

Table S5. Primers used in qRT-PCR of miRNAs.

Primer	Sequence (5'-3')
miR160-stemloop	GTCGTATCCAGTGCAGGGTCCGAGGTATTCGCACTGGATACGACTGGCAT
miR166-stemloop	GTCGTATCCAGTGCAGGGTCCGAGGTATTCGCACTGGATACGACGGGGAA
miR319-stemloop	GTCGTATCCAGTGCAGGGTCCGAGGTATTCGCACTGGATACGACTGAATG
miR390-stemloop	GTCGTATCCAGTGCAGGGTCCGAGGTATTCGCACTGGATACGACGGCGCT
miR396-stemloop	GTCGTATCCAGTGCAGGGTCCGAGGTATTCGCACTGGATACGACCAGTTC
miR162-stemloop	GTCGTATCCAGTGCAGGGTCCGAGGTATTCGCACTGGATACGACCTGGAT
miR168-stemloop	GTCGTATCCAGTGCAGGGTCCGAGGTATTCGCACTGGATACGACGTCCCG
miR160-qF	AACAATTGCCTGGCTCCCT
miR166-qF	AACGCACGGAATGTTGTCTG
miR319-qF	AACCACTAGCTGCCGAATCA
miR390-qF	AACAGTGAAGCTCAGGAGGG
miR396-qF	AACACGCTTCCACAGCTTTC
miR162-qF	ACCACCGTCGATAAACCTCT
miR168-qF	AACAAGTCGCTTGGTGCAG
smal RNA universal primer R	GTCGTATCCAGTGCAGGGT

Table S6. Primers used in qRT-PCR of Pri-miRNAs, miRNA target genes, and miRNA processing-related genes.

Marker	Forward primer (5′–3′)	Reverse primer (5′–3′)
U6	CGATAAAATTGGAACGATACAGA	ATTTGGACCATTTCTCGATTTGT
Pri-miRNA160	GCCTCCATCGAGCAATCAGA	TACACACGCACACCACACAA
Pri-miRNA166	TGTTGTCTGGTTCAAGGTCTCA	GAGGGGAATGAAGCCTGGTC
Pri-miRNA319	CACCAGGATGAGGCAGCAAA	GTAGCCTTAGCAGGGTTTCCA
Pri-miRNA390	CAGGCTGCTGATCTGAGAGA	GCCATCAACAAGCACGAACA
Pri-miRNA396	CCTAAAACGGTGGCTGTGGA	TGTGCCCTTCATTGTCTGGA
Osubi1	CGCACCCTTGCGGATT	TACTGGCCACCACGGAG
OsARF18	TGGAGGAGTTCCATTGTGCC	AGAGAGATCAAGTGTGCGCC
OsHB1	AAGTGGTGTATGCCTTGGGG	CCGCCCATTATAGACCACCC
OsHB2	CAATGGGTCTTCTGGGCCAT	CTGATCCATGTACGACGCGA
OsHB3	CAGTTCTGCATCCGCAAAGG	CCCATTCGACAGCAGTTCCT
OsHB4	ATTTCGTTGCTTGATCGCCC	TCAGGCCGTGCTTACAACAA
OsHB5	ATGCCACTTGTCCAGCCTTT	ATGTGTATCACGCTGCCTCC
PCF5	ACACCGCCATCCAGTTCTAC	CGTCCTTGGCGTTCTTGATG
PCF6	ATGTCCTCGGGTTTCTTGGG	GAAGGAAATGGTGGAGCCGT
PCF7	GTCAAGTCCCTGTTCCCCTC	GTTCGACTGTAGGGTGAGGC
PCF8	CTCCACTGCTCGAACACCAA	ATCACCTCGAACGACATGGG
TCP21	TCGACGCGTGTCAACTACC	GCGGTTGTACGCATTGAGC
ARF3	TGGTGGTGGAATCGAAAGGG	TGTGCTTGTATCAGAGGCCG
SRL1	TCCTCATCTCCTGCCTCCTC	GAACCAAGGGGTTGGGAGAG
DCL1	TGCCTGACAGTCATGGTGAG	TGATCAAAGCCTGCACCCTA
DRB1-2	GAGGCATCTTGTGGCACTCT	AGCGTCACTGTGTTGGTTCT
DLN175	ATGGAACTCTGGACCTTGGC	TCTTTTGAGGAACCCCTGCC
WAF1	GCTTGAGCATCCAACAACCC	CACAGCCGTTGGAACACTTG
AGO1	AGACTCGCTTGCCATTTCTGT	ACAGTGGTAGCACGAGTAGG
DRB2	GTGGTCTGCTTTTACCCCCA	GCATGGCAAAGCAGGAGTAT
OsActin	TGTATGCCAGTGGTCGTACCA	CCAGCAAGGTCGAGACGAA

Table S7. Primers used in qRT-PCR of the genes related to leaf shape and cell growth.

Marker	Forward primer (5′–3′)	Reverse primer (5′–3′)	Reference
ACL1	GACCCCGAGCAATTCTACCC	GTAACCCACTTGCTGTTGCG	
NRL1	AAGAGGGACTTCCTCAAGAACAAG	TCGTACTCGCGCTTCACCTT	
REL2	TTGCCTTGTTGGGAGTTGGT	TGCACTTCACAGGACAAGCA	
ROC5	ATGAATGAGTTCGCCGGAGG	CGGACCTGTCGATGTTACCC	
REL1	GCACTCCAGCTTGGAACTCT	TGTTGTTGGCGTAGCTGAGT	
RLI4	GAACCGTAGCCATCGACCAT	GTTCAACGCAGTTTACAGGC	
ZHD1	CAGTTCTGTGACGAGGTCGG	ATGCATTTGCTCCCGGTCAT	
OsCycD2	TCCTCTCCGTGTTCGATCTTCCTC	CAGCCAAAGACAAGGAAGCTACTG	[39]
OsCycD4	ATCTTCGCGTCAGGATGGATGC	AGCTGTAGTAGGAGTGAACCTTCC	[39]
OsH4	TTGCCCTGTGTAGATTGCTG	GCAACAAGGTGAATTTCAAACA	[39]
OsEXPA	TGATCATAACGTACGCTACGCA	CAAAACACATTCATTATTCGCCA	[39]
OsXTR	AGGTGGTGTCTTGTAACTTTTGTTGTTA	ATGGTCCCAAAAGCACAAGAGT	[39]
